# Evaluating the Relative Environmental Impact of Countries

**DOI:** 10.1371/journal.pone.0010440

**Published:** 2010-05-03

**Authors:** Corey J. A. Bradshaw, Xingli Giam, Navjot S. Sodhi

**Affiliations:** 1 The Environment Institute and School of Earth & Environmental Sciences, University of Adelaide, Adelaide, South Australia, Australia; 2 South Australian Research and Development Institute, Henley Beach, South Australia, Australia; 3 Department of Biological Sciences, National University of Singapore, Singapore, Singapore; 4 Department of Ecology and Evolutionary Biology, Princeton University, Princeton, New Jersey, United States of America; 5 Department of Organismic and Evolutionary Biology, Harvard University, Cambridge, Massachusetts, United States of America; University of Durham, United Kingdom

## Abstract

Environmental protection is critical to maintain ecosystem services essential for human well-being. It is important to be able to rank countries by their environmental impact so that poor performers as well as policy ‘models’ can be identified. We provide novel metrics of country-specific environmental impact ranks – one proportional to total resource availability per country and an absolute (total) measure of impact – that explicitly avoid incorporating confounding human health or economic indicators. Our rankings are based on natural forest loss, habitat conversion, marine captures, fertilizer use, water pollution, carbon emissions and species threat, although many other variables were excluded due to a lack of country-specific data. Of 228 countries considered, 179 (proportional) and 171 (absolute) had sufficient data for correlations. The proportional index ranked Singapore, Korea, Qatar, Kuwait, Japan, Thailand, Bahrain, Malaysia, Philippines and Netherlands as having the highest proportional environmental impact, whereas Brazil, USA, China, Indonesia, Japan, Mexico, India, Russia, Australia and Peru had the highest absolute impact (i.e., total resource use, emissions and species threatened). Proportional and absolute environmental impact ranks were correlated, with mainly Asian countries having both high proportional and absolute impact. Despite weak concordance among the drivers of environmental impact, countries often perform poorly for different reasons. We found no evidence to support the environmental Kuznets curve hypothesis of a non-linear relationship between impact and per capita wealth, although there was a weak reduction in environmental impact as per capita wealth increases. Using structural equation models to account for cross-correlation, we found that increasing wealth was the most important driver of environmental impact. Our results show that the global community not only has to encourage better environmental performance in less-developed countries, especially those in Asia, there is also a requirement to focus on the development of environmentally friendly practices in wealthier countries.

## Introduction

The environmental crises currently gripping the planet [1], [2] are the corollary of excessive human consumption of natural resources [3]. Indeed, there is considerable and mounting evidence that elevated degradation and loss of habitats and species are compromising ecosystem services that sustain the quality of life for billions of people worldwide [1], [4], [5]. Continued degradation of nature despite decades of warning [1], coupled with the burgeoning human population (currently estimated at nearly 7 billion and projected to reach 9–10 billion by 2050) [1], [6], suggest that human quality of life could decline substantially in the near future. Increasing competition for resources could therefore lead to heightened civil strife and more frequent wars [7]. Continued environmental degradation demands that countries needing solutions be identified urgently so that they can be assisted in environmental conservation and restoration. Identifying those nations whose policies have managed successfully to reduce environmental degradation should be highlighted to inspire other nations to achieve better environmental outcomes for their own long-term prosperity.

Policy makers require good information on which to base their decisions to reduce environmental degradation and restore ecosystems [8]. In the spirit of minimizing carbon emissions [9], environmental performance can be measured via international rankings to provide benchmarks against which improvements can be assessed [8]. Many such rankings exist, such as the City Development Index (CDI), Ecological Footprint (EF), Environmental Performance Index (EPI), Environmental Sustainability Index (ESI), Genuine Savings Index (GSI), Human Development Index (HDI), Living Planet Index (LPI), and the Well-Being Index (WI) [reviewed in 8]. However, all such indices have problems associated with their inability to describe the complexity of ‘sustainable’ development, lack of comprehensiveness, and arbitrary or subjective assumptions regarding normalization and weighting [8]. Most indices also incorporate (often arbitrarily) indicators of human health and economic performance, so the emphasis on the environmental component *per se* is diluted or confounded. Indeed, each set of criteria used to rank nations depends on the particular goal of the ranking itself, the assumptions associated with the data (i.e., precision, robustness, extent), and the hypotheses posed to explain among-nation trends.

Economists and social scientists have attempted to explain trends among countries for various indices of environmental performance based primarily on human population density, wealth and governmental structure and efficacy, with varying results. Perhaps the most controversial is the environmental Kuznets curve (EKC) hypothesis [10] and the theory of ecological modernization [11] which argue that environmental performance and per capita wealth follow a U-shaped relationship among countries. In other words, instead of higher environmental impact associated with increasing wealth and the corollary of higher per capita resource consumption [12]–[15], the EKC predicts that beyond a certain threshold, wealthier societies can reduce environmental degradation via cleaner technologies and higher demand for sustainable behavior from their citizenry [10]. This evidence for the EKC hypothesis is equivocal; some analyses suggest that measures of environmental degradation (i.e., deforestation [16], air and water pollution [11], and number of threatened birds and mammals [17], [18]) increase initially with economic growth, but then decline after a threshold. However, others suggest that increasing economic development leads to higher species endangerment [11], [17], and general levels of species threat [19]. There is also evidence for an interaction between a country's wealth and its rate of deforestation or afforestation – poor countries with little forest cover consume that remaining portion more quickly than do poor countries containing relatively more forests [20].

In addition to metrics of wealth, other indices of socio-economic performance such as human population size and density, and governance quality correlate with environmental performance measures [15], [21]–[25]. Indeed, human population density is positively associated with the number of threatened species per country [11], [18]. Although the influence of governance type and quality on environmental performance is still hotly debated [25], political corruption (the ‘unlawful use of public office for private gain’) [23], [24] is expected to erode environments and exacerbate biodiversity loss [23]. Corruption has been linked to deforestation [20], [25], [26], CO_2_/NOx emissions, land degradation, organic pollution in water [25] and an index of environmental ‘sustainability’ [27], although others have not found evidence for a relationship between change in natural forest cover and mean governance scores [23].

One of the principal reasons results are inconsistent and the relationship continues to be debated is that the importance of different correlates varies among regions [28], and there are many different methods used and assumptions made regarding metrics and exceptions [8], [11], [15], [25]. Our goal here is to provide a set of simple, yet novel metrics of environmental impact that rank countries by their proportional (relative to resource availability per country) and absolute (total degradation as measured by different environmental metrics) resource consumption, deforestation, pollution and biodiversity loss. These metrics are intended to improve policy and practice in the regions identified as having the poorest environmental performance so that global benefits will arise; we contend that the beneficiaries of policies that our ranking system could influence would be global in extent, such as international trade treaties, carbon taxation, and development aid. We use the unit of ‘country’ as a basis for environmental impact rank because a government's decisions affecting the state of the environment can be realistically best made at this level [29]. We provide different rankings that combine important (and readily available) variables of past and current environmental impact (forest loss, natural habitat conversion to managed/crop/urban uses, marine captures, fertilizer use, water pollution, carbon emissions and species threat), but do not confound environmental performance with indicators of human health (e.g., EPI) or economics (e.g., GSI). Our indices are also transparently and objectively constructed, and are particularly robust to the inclusion/exclusion of component metrics.

Specifically, we aimed to: (*i*) provide a rank of proportional environmental impact to determine how countries perform with respect to their available resources, (*ii*) provide a rank of total (absolute) resource use to determine which countries have the highest (and lowest) impact at the global scale, (*iii*) examine concordance among the different measures of environmental impact within our composite indices to test whether a country's performance is uniform across the environmental spectrum, (*iv*) determine the correlation between our environmental impact ranks and existing indices of environmental performance; (*v*) test for correlations between environmental impact ranks and those associated with population size, governance quality and wealth using non-parametric, parametric and structural equation models, and (*vi*) test the EKC hypothesis that relative environmental impact is nonlinearly related to per-capita wealth.

## Results

### Proportional rank

**Figure 1:**
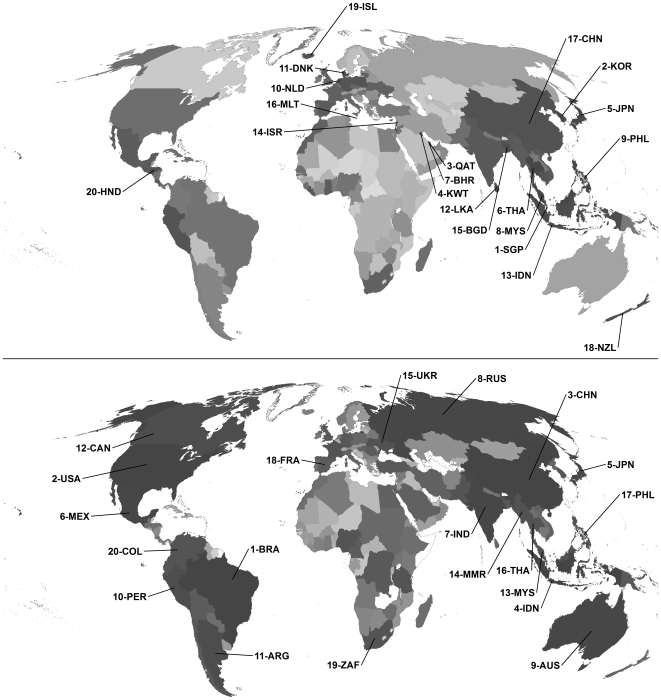
Relative rank of countries by proportional and absolute environmental impact. Proportional environmental impact (179 countries; top panel) and absolute environmental impact rank (171 countries; bottom panel) (darker grey = higher impact) out of 228 countries considered are shown. Environmental impact ranks (proportional and absolute) combine ranks for natural forest lost, habitat conversion, marine captures, fertilizer use, water pollution, carbon emissions and proportion of threatened species (see text for details). The worst 20 countries (codes described in Tables 1 & 2) for each ranking are shown.

Using the constraint of missing no more than three values within the composite environmental impact rank (see File S1 and Tables S1, S2, S3, S4 for sensitivity analysis of this choice), there were 179 countries out of the entire dataset of 228 for which sufficient data were available for correlation analyses (a list of the 49 missing countries [mostly small island nations] is provided in Table S5). This index ranked the following 10 countries as having the highest proportional environmental impact: Singapore, Korea, Qatar, Kuwait, Japan, Thailand, Bahrain, Malaysia, Philippines and the Netherlands (Table 1; Fig. 1, top panel). The 10 lowest proportional impact countries were Eritrea, Suriname, Lesotho, Turkmenistan, Gabon, Kazakhstan, Mali, Vanuatu, Chad and Bhutan (Table 2). The full proportional ranking of all 179 countries is provided in Table S6. Proportional rankings were robust to the inclusion/exclusion of composite metrics – removing each component metric one at a time and recalculating the proportional rank maintained the general characteristics of the ranking (Kendall's *τ* = ranged from 0.799 to 0.877 between the original and modified ranks; Table 3).

**Table 1:** Twenty worst-ranked countries by proportional composite environmental (pENV) rank (lower ranks = higher negative impact).

Rank	Country	Code	PD	PGR	GOV	GNI	NFL	HBC	MC	FER	WTP	PTHR	CO2	pENV
1	Singapore	SGP	1	51	13	115	**128**	**5**	**91**	**1**	**4**	**63**	**1**	10.6
2	Rep Korea	KOR	14	158	56	154	**23**	**61**	**20**	**17**	**21**	**29**	**5**	20.4
3	Qatar	QAT	108	8	67	-	**-**	**198**	**112**	**20**	**3**	**-**	**7**	24.8
4	Kuwait	KWT	61	110	74	109	**128**	**197**	**114**	**11**	**1**	**-**	**8**	25.1
5	Japan	JPN	23	188	30	165	**87**	**87**	**18**	**21**	**29**	**13**	**6**	25.2
6	Thailand	THA	71	145	90	148	**43**	**8**	**7**	**67**	**-**	**37**	**46**	25.5
7	Bahrain	BHR	6	41	73	52	**-**	**193**	**59**	**4**	**-**	**123**	**2**	25.7
8	Malaysia	MYS	102	60	71	131	**47**	**75**	**22**	**8**	**77**	**15**	**11**	25.9
9	Philippines	PHL	36	70	122	144	**22**	**20**	**48**	**57**	**70**	**3**	**38**	26.7
10	Netherlands	NLD	16	166	9	151	**171**	**25**	**11**	**12**	**-**	**173**	**4**	27.0
11	Denmark	DNK	70	181	3	125	**178**	**4**	**12**	**52**	**9**	**180**	**16**	27.4
12	Sri Lanka	LKA	34	156	110	111	**31**	**56**	**33**	**30**	**41**	**7**	**34**	28.9
13	Indonesia	IDN	74	118	153	153	**5**	**76**	**62**	**59**	**79**	**12**	**14**	29.3
14	Israel	ISR	33	40	64	123	**128**	**110**	**93**	**5**	**6**	**62**	**9**	30.0
15	Bangladesh	BGD	5	80	166	134	**84**	**1**	**26**	**45**	**81**	**36**	**101**	31.2
16	Malta	MLT	4	154	21	36	**-**	**214**	**127**	**69**	**2**	**138**	**3**	34.0
17	China	CHN	64	149	129	166	**194**	**111**	**3**	**29**	**33**	**20**	**47**	34.5
18	New Zealand	NZL	177	128	6	113	**98**	**89**	**73**	**13**	**91**	**1**	**93**	35.4
19	Iceland	ISL	207	144	2	44	**128**	**195**	**13**	**2**	**106**	**-**	**-**	36.9
20	Honduras	HND	124	66	135	76	**1**	**39**	**125**	**82**	**72**	**44**	**75**	37.0

Shown are country names and codes (see also Fig. 1), population density (PD) rank, population growth rate (PGR) rank, governance quality (GOV) rank, Gross National Income (GNI) rank, natural forest loss (NFL) rank, natural habitat conversion (HBC) rank, marine captures (MC) rank, fertilizer use (FER) rank, water pollution (WTP) rank, proportion of threatened species (PTHR) rank, and carbon emissions (CO2) rank. Constituent variables used to create the pENV are in boldface. See text for details. Missing values denoted by ‘-’.

**Table 2:** Twenty top-ranked countries by proportional composite environmental (pENV) rank (higher ranks = lower negative impact).

Rank	Country	Code	PD	PGR	GOV	GNI	NFL	HBC	MC	FER	WTP	PTHR	CO2	pENV
179	Cape Verde	CPV	69	54	76	20	**128**	**214**	**113**	**157**	**-**	**-**	**-**	148.5
178	Cent Afr Rep	CAF	199	67	188	29	**76**	**172**	**176.5**	**174**	**-**	**175**	**131**	144.8
177	Swaziland	SWZ	116	96	142	31	**201**	**192**	**176.5**	**113**	**67**	**167**	**148**	143.9
176	Antig & Barb	ATG	50	85	52	9	**128**	**148**	**119**	**-**	**-**	**176**	**-**	141.1
175	Niger	NER	191	10	143	46	**80**	**178**	**176.5**	**173**	**109**	**128**	**145**	136.4
174	Grenada	GRD	30	164	66	6	**128**	**214**	**115**	**-**	**-**	**109**	**-**	136.1
173	Samoa	WSM	117	150	65	14	**196**	**214**	**95**	**96**	**-**	**-**	**116**	134.7
172	Tonga	TON	66	185	109	8	**128**	**214**	**132**	**-**	**-**	**-**	**88**	133.6
171	Djibouti	DJI	153	53	151	19	**128**	**184**	**152**	**-**	**-**	**98**	**109**	130.8
170	Tajikistan	TJK	137	119	182	38	**161**	**124**	**176.5**	**111**	**-**	**93.5**	**-**	129.6
169	Bhutan	BTN	183	143	81	-	**198**	**85**	**176.5**	**169**	**-**	**53**	**142**	124.8
168	Chad	TCD	197	12	181	41	**70**	**112**	**176.5**	**148**	**-**	**125**	**144**	124.3
167	Vanuatu	VUT	172	48	88	4	**128**	**165**	**81**	**-**	**-**	**-**	**139**	124.2
166	Mali	MLI	193	29	103	50	**65**	**114**	**176.5**	**137**	**-**	**148**	**137**	124.0
165	Kazakhstan	KAZ	200	207	146	114	**157**	**107**	**176.5**	**152**	**-**	**57**	**-**	120.8
164	Gabon	GAB	201	63	125	39	**81**	**161**	**86**	**163**	**110**	**144**	**124**	120.0
163	Turkmenistan	TKM	192	91	189	70	**128**	**182**	**176.5**	**90**	**-**	**66**	**-**	119.6
162	Lesotho	LSO	114	116	102	34	**128**	**126**	**176.5**	**120**	**46**	**157**	**138**	119.1
161	Suriname	SUR	208	152	94	22	**128**	**181**	**66**	**73**	**-**	**183**	**136**	118.6
160	Eritrea	ERI	148	52	168	27	**77**	**117**	**148**	**133**	**-**	**132**	**-**	118.5

Shown are country names and codes, population density (PD) rank, population growth rate (PGR) rank, governance quality (GOV) rank, Gross National Income (GNI) rank, natural forest loss (NFL) rank, natural habitat conversion (HBC) rank, marine captures (MC) rank, fertilizer use (FER) rank, water pollution (WTP) rank, proportion of threatened species (PTHR) rank, and carbon emissions (CO2) rank. Constituent variables used to create the pENV are in boldface. See text for details. Missing values denoted by ‘-’.

**Table 3:** Ten worst- and best-ranked countries by proportional composite rank (pENV) with each of the 7 composite metrics removed sequentially (i.e., pENV calculated from 6 metrics only).

	pENV	exNFL	exHBC	exMC	exFER	exWTP	exTHR	exCO2
	***τ*** ** = **	0.799	0.812	0.803	0.807	0.864	0.800	0.877
**10 Worst**								
	Singapore	**Singapore**	**Singapore**	**Singapore**	**Singapore**	**Singapore**	**Singapore**	**Singapore**
	Rep Korea	**Kuwait**	**Qatar**	**Qatar**	Taiwan	Taiwan	**Bahrain**	Taiwan
	Qatar	**Netherlands**	**Bahrain**	**Kuwait**	**Rep Korea**	**Rep Korea**	**Netherlands**	**Thailand**
↑	Kuwait	Taiwan	**Kuwait**	**Rep Korea**	**Thailand**	**Malaysia**	**Rep Korea**	**Philippines**
*worse*	Japan	**Rep Korea**	**Rep Korea**	**Bahrain**	**Philippines**	**Philippines**	Denmark	Bangladesh
	Thailand	Denmark	**Japan**	**Philippines**	Denmark	**Japan**	**Thailand**	Rep Korea
	Bahrain	**Japan**	**Malaysia**	Israel	**Japan**	Indonesia	**Qatar**	Sri Lanka
	Malaysia	**Thailand**	Malta	Indonesia	Indonesia	**Thailand**	**Kuwait**	**Malaysia**
	Philippines	**Malaysia**	Israel	Malta	**Qatar**	**Bahrain**	Malta	Denmark
	Netherlands	Israel	Iceland	**Malaysia**	Sri Lanka	Bangladesh	Israel	New Zealand
**10 Best**								
	Tajikistan	Guinea-Bissau	Chad	Fr Guiana	**Antig & Barb**	Lesotho	**Swaziland**	**Samoa**
	Djibouti	Martinique	**Niger**	Vanuatu	**Cape Verde**	**Antig & Barb**	**Djibouti**	**Antig & Barb**
	Tonga	**Antig & Barb**	**Tajikistan**	**Grenada**	**Samoa**	**Niger**	**Tajikistan**	**Swaziland**
	Samoa	Niger	**Cape Verde**	**Samoa**	New Caledonia	**Cen Afr Rep**	Kazakhstan	**Cen Afr Rep**
	Grenada	Marshall Is	Bhutan	**Antig & Barb**	**Swaziland**	**Cape Verde**	**Grenada**	**Cape Verde**
*better*	Niger	**Cape Verde**	**Swaziland**	Cayman Is	Cayman Is	Cayman Is	Bhutan	**Tonga**
↓	Antig & Barb	Fr Guiana	**Atig & Barb**	Martinique	Fr Polynesia	Marshall Is	**Cape Verde**	Cayman Is
	Swaziland	**Cen Afr Rep**	**Cen Afr Rep**	**Cape Verde**	Bermuda	**Swaziland**	Marshall Is	Marshall Is
	Cent Afr Rep	Andorra	Andorra	Andorra	Andorra	Andorra	Andorra	Andorra
	Cape Verde	Liechtenstein	Liechtenstein	Liechtenstein	Liechtenstein	Liechtenstein	Liechtenstein	Liechtenstein

Metrics excluded (ex) sequentially include proportional natural forest loss (NFL), proportional natural habitat conversion (HBC), proportional marine captures (MC), proportional fertilizer use (FER), proportional water pollution (WTP), proportion of threatened species (THR), and proportional carbon emissions (CO2). See Materials and Methods for calculation of proportional metrics. Boldface cells indicate which countries appeared in the full pENV (calculated from 7 composite metrics) for each reduced pENV (i.e., with one metric removed). For each reduced pENV, Kendall's *τ* correlation to the full pENV is shown (all P<0.0001 and *n* = 179 countries).

### Absolute rank

Based again on no more than three missing values, the absolute composite ranking could be calculated for 171 countries (57 missing countries provided in Table S7). The full absolute ranking of all 171 countries is provided in Table S8. From a global perspective, the most populous and economically influential countries generally had the highest absolute environmental impact: Brazil, USA, China, Indonesia, Japan, Mexico, India, Russia, Australia and Peru were the 10 worst-ranked countries (Table 4; Fig. 1, bottom panel). The absolute and proportional environmental impact ranks were negatively correlated (Kendall's *τ* = −0.28, P = 0.0001, *n* = 170 countries); but China, Indonesia, Japan, Malaysia, Thailand, and Philippines appeared in the list of highest-impact countries for both proportional and absolute ranks (Tables 2 & 4). Absolute rankings were even more robust to the inclusion/exclusion of composite metrics (Kendall's *τ* = ranged from 0.808 to 0.929 between the original and modified ranks; Table 5).

**Table 4:** Twenty worst-ranked countries by absolute composite environmental (aENV) rank (lower ranks = higher negative impact).

Rank	Country	Code	PD	PGR	GOV	GNI	NFL	HBC	MC	FER	WTP	THR	CO2	aENV
1	Brazil	BRA	166	114	95	159	**1**	**3**	**30**	**3**	**8**	**4**	**4**	4.5
2	USA	USA	156	139	20	167	**21**	**211.5**	**3**	**1**	**2**	**9**	**1**	5.9
3	China	CHN	64	149	129	166	**216**	**36**	**1**	**-**	**1**	**6**	**2**	6.7
4	Indonesia	IDN	74	118	153	153	**2**	**183**	**6**	**6**	**7**	**3**	**3**	7.0
5	Japan	JPN	23	188	30	165	**73**	**5**	**4**	**17**	**5**	**23.5**	**6**	10.8
6	Mexico	MEX	131	115	93	156	**9**	**211.5**	**17**	**13**	**17**	**1**	**12**	13.6
7	India	IND	21	90	106	164	**214**	**137**	**8**	**2**	**3**	**8**	**8**	13.7
8	Russia	RUS	194	202	141	158	**12**	**125**	**7**	**18**	**4**	**26**	**5**	13.9
9	Australia	AUS	209	127	11	152	**10**	**7**	**47**	**9**	**31**	**11.5**	**18**	15.2
10	Peru	PER	168	111	120	119	**27**	**30**	**2**	**46**	**49**	**7**	**27**	18.3
11	Argentina	ARG	181	134	121	149	**19**	**11**	**21**	**23**	**22**	**16**	**31**	19.6
12	Canada	CAN	204	141	10	155	**133.5**	**6**	**19**	**7**	**16**	**71**	**10**	19.8
13	Malaysia	MYS	102	60	71	131	**39**	**170**	**16**	**22**	**24**	**10**	**9**	24.3
14	Myanmar	MMR	111	132	197	-	**4**	**18**	**22**	**113**	**102**	**25**	**14**	25.2
15	Ukraine	UKR	103	208	137	141	**201**	**1**	**39**	**36**	**11**	**90**	**-**	25.6
16	Thailand	THA	71	145	90	148	**28**	**211.5**	**9**	**11**	**-**	**20**	**29**	26.4
17	Philippines	PHL	36	70	122	144	**22**	**168**	**12**	**27**	**21**	**11.5**	**33**	26.6
18	France	FRA	79	172	24	161	**210**	**-**	**26**	**4**	**9**	**116.5**	**16**	26.7
19	South Africa	ZAF	147	93	72	147	**63**	**43**	**25**	**28**	**19**	**31**	**17**	29.4
20	Colombia	COL	146	102	138	139	**43**	**162**	**64**	**30**	**30**	**2**	**32**	30.7

Shown are country names and codes (see also Fig. 1), population density (PD) rank, population growth rate (PGR) rank, governance quality (GOV) rank, Gross National Income (GNI) rank, natural forest loss (NFL) rank, natural habitat conversion (HBC) rank, marine captures (MC) rank, fertilizer use (FER) rank, water pollution (WTP) rank, threatened species (THR) rank, and carbon emissions (CO2) rank. Constituent variables used to create the aENV are in boldface. See text for details. Missing values denoted by ‘-’.

**Table 5:** Ten worst- and best-ranked countries by absolute composite rank (aENV) with each of the 7 composite metrics removed sequentially (i.e., aENV calculated from 6 metrics only).

	aENV	exNFL	exHBC	exMC	exFER	exWTP	exTHR	exCO2
	***τ*** ** = **	0.816	0.808	0.890	0.904	0.929	0.870	0.917
**10 Worst**								
	Brazil	**China**	**USA**	**Brazil**	**Brazil**	**Brazil**	**Brazil**	**Brazil**
	USA	**USA**	**Indonesia**	**USA**	**China**	**Indonesia**	**USA**	**USA**
	China	**Brazil**	**Brazil**	**Indonesia**	**Indonesia**	**USA**	**China**	**Indonesia**
↑	Indonesia	**Japan**	**China**	**China**	**USA**	**China**	**Indonesia**	**China**
*worse*	Japan	**Indonesia**	**Mexico**	**Australia**	**Japan**	**Japan**	**Japan**	**Japan**
	Mexico	**India**	**India**	**Japan**	**Russia**	**Mexico**	**Russia**	**Mexico**
	India	Canada	**Russia**	**Mexico**	**Mexico**	**Australia**	Zambia	**Australia**
	Russia	**Russia**	**Japan**	**India**	**Peru**	**Peru**	**India**	**India**
	Australia	**Mexico**	**Peru**	**Russia**	**Australia**	**Russia**	Canada	**Russia**
	Peru	**Australia**	**Australia**	Argentina	Argentina	**India**	**Australia**	**Peru**
**10 Best**								
	Tonga	**Grenada**	**St Lucia**	Cayman Is	St Helena	Cayman Is	Bermuda	**Djibouti**
	St Kitts & Nevis	Timor-Leste	**Antig & Barb**	Fr Polynesia	Martinique	**St Lucia**	St Helena	Martinique
	Gambia	Bermuda	Gibraltar	Neth Antilles	Cayman Is	Amer Samoa	Martinique	Cayman Is
	St Vincent Gren	US Virgin Is	Br Virgin Is	**St Lucia**	**St Lucia**	**Grenada**	**Antig & Barb**	**St Lucia**
	Swaziland	Martinique	Montserrat	Vanuatu	Amer Samoa	**Antig & Barb**	**Djibouti**	Amer Samoa
*better*	Barbados	Cayman Is	**St Kitts & Nevis**	**Antig & Barb**	**Antig & Barb**	Palestine	**St Lucia**	**Antig & Barb**
↓	Djibouti	**St Lucia**	Anguilla	Montserrat	Palestine	Bermuda	Amer Samoa	Palestine
	Grenada	**Antig & Barb**	Cayman Is	Martinique	Andorra	Andorra	Palestine	Andorra
	St Lucia	Montserrat	Palau	Andorra	Montserrat	Montserrat	Anguilla	Montserrat
	Antig & Barb	Anguilla	Monaco	Anguilla	Anguilla	Anguilla	Montserrat	Anguilla

Metrics excluded (ex) sequentially include natural forest loss (NFL), natural habitat conversion (HBC), marine captures (MC), fertilizer use (FER), water pollution (WTP), threatened species (THR), and carbon emissions (CO2). Boldface cells indicate which countries appeared in the full aENV (calculated from 7 composite metrics) for each reduced aENV (i.e., with one metric removed). For each reduced aENV, Kendall's *τ* correlation to the full aENV is shown (all P<0.0001 and *n* = 171 countries).

### Concordance among environmental variables

There was modest concordance among the individual proportional environmental variable rankings (Table 1) making up the proportional composite ranking (Kendall's *W* = 0.26, P<0.0001). This demonstrates that environmental impact in one aspect is partially mirrored by impact in other measures presumably because high urbanization leads to higher proportional natural forest loss, greater release of CO_2_ through land-use change and burning of fossil fuels, and an ensuing higher proportion of species threatened with extinction owing to habitat loss and pollution. Despite this moderate concordance, countries can perform poorly for somewhat different reasons (Tables 6 & 7); for example, Singapore, Bahrain and Malta had high relative fertilizer use and CO_2_ emissions, Indonesia and Honduras had high rates of deforestation, Bangladesh and Denmark had high habitat conversion, China had high marine captures, and New Zealand had a high proportion of threatened species (Tables 1, 6, 7).

**Table 6:** Ten worst- and best-ranked countries by proportional environmental metrics: proportional natural forest loss (NFL), proportional natural habitat conversion (HBC), proportional marine captures (MC), proportional fertilizer use (FER), proportional water pollution (WTP), proportion of threatened species (THR), and proportional carbon emissions (CO2).

	NFL^1^	HBC^2^	MC^2^	FER	WTP	THR	CO2
**10 Worst**							
	Honduras	Bangladesh	Peru	Singapore	Kuwait	New Zealand	Singapore
	Solomon Is	N Mariana Is	Taiwan	Iceland	Malta	Madagascar	Bahrain
	Guam	Monaco	China	St Lucia	Qatar	Philippines	Malta
↑	DPR Korea	Denmark	Ghana	Bahrain	Singapore	Mexico	Netherlands
*worse*	Indonesia	Singapore	Morocco	Israel	Bahamas	Haiti	Rep Korea
	Micronesia	Hungary	Lithuania	Dominica	Israel	Cuba	Japan
	Cambodia	Jersey	Thailand	Costa Rica	Barbados	Sri Lanka	Qatar
	Timor-Leste	Thailand	Togo	Malaysia	Jordan	Seychelles	Kuwait
	Zimbabwe	El Salvador	Namibia	St Vincent Gren	Denmark	Sao Tome Princ	Israel
	Niue	Sierra Leone	Senegal	UAE	Czech Rep	Dominican Rep	Germany
	Ecuador	San Marino	Netherlands	Kuwait	Tunisia	Fiji	Malaysia
**10 Best**							
	Bhutan	Libya	Sudan	Namibia	Colombia	Belgium	Burkina Faso
	Slovenia	Jordan	Fr Polynesia	Marshall Is	Mozambique	Denmark	Mongolia
	Palau	Swaziland	Bermuda	Uganda	Cameroon	Fr Guiana	Bhutan
	Swaziland	Bahrain	Guinea-Bissau	Afghanistan	Iceland	Sweden	Namibia
	Cuba	Saudi Arabia	Eritrea	Bhutan	Peru	Suriname	Chad
*better*	Viet Nam	Iceland	Aruba	Somalia	Bolivia	Cayman Is	Niger
↓	Italy	UAE	Bahamas	Rwanda	Niger	Anguilla	Gambia
	Liechtenstein	Kuwait	New Caledonia	Dem Rep Congo	Gabon	Liechtenstein	Viet Nam
	Spain	Qatar	Djibouti	Niger	Myanmar	Luxembourg	Swaziland
	Austria	Greenland	Bosnia/Herz	Cen Afr Rep	Afghanistan	San Marino	Uruguay

See Materials and Methods for calculation of proportional metrics.

1 10 Best countries have reported increasing forest cover.

2 Countries with zero proportional habitat conversion/marine captures excluded.

**Table 7:** Ten worst- and best-ranked countries by individual absolute environmental metrics: natural forest loss (NFL), natural habitat conversion (HBC), marine captures (MC), fertilizer use (FER), water pollution (WTP), total threatened species (THR), and carbon emissions (CO2).

	NFL^1^	HBC^2^	MC^2^	FER	WTP	THR^2^	CO2
**10 Worst**							
	Brazil	Ukraine	China	USA	China	Mexico	USA
	Indonesia	Romania	Peru	India	USA	Columbia	China
	Sudan	Brazil	USA	Brazil	India	Indonesia	Indonesia
↑	Myanmar	Japan	Japan	France	Russia	Brazil	Brazil
*worse*	Dem Rep Congo	Canada	Chile	Pakistan	Japan	Ecuador	Russia
	Zambia	Australia	Indonesia	Indonesia	Germany	China	Japan
	Nigeria	Hungary	Russia	Canada	Indonesia	Peru	Germany
	Tanzania	Tanzania	India	Germany	Brazil	India	India
	Mexico	Trin & Tobago	Thailand	Australia	France	USA	Malaysia
	Australia	Argentina	Norway	Viet Nam	UK	Malaysia	Canada
**10 Best**							
	Greece	Mauritania	N Mariana Is	Namibia	Burkina Faso	Denmark	Lesotho
	Belarus	Grenada	Tokelau	Burundi	Barbados	Estonia	Samoa
	Cuba	San Marino	Aruba	Barbados	Swaziland	St Kitts/Nevis	Tonga
	France	Djibouti	Jordan	Eritrea	Gabon	Br Virgin Is	Sao Tome/Princ
	Austria	Ghana	Cayman Is	Gabon	Bahamas	Antigua/Barb	Vanuatu
*better*	Italy	Rep Korea	Nauru	Malta	Niger	Sweden	Cook Is
↓	Viet Nam	St Lucia	Montserrat	Fr Polynesia	Grenada	Andorra	Gambia
	India	Andorra	Pitcairn I	Maldives	Bermuda	Cayman Is	Swaziland
	Spain	Bahrain	Bosnia/Herz	Samoa	St Vincent Gren	Monaco	Viet Nam
	China	Fr Polynesia	Monaco	Marshall Is	Afghanistan	Anguilla	Uruguay

1 10 Best countries have reported increasing forest cover.

2 Countries with zero habitat conversion/marine captures/threatened species excluded.

For the absolute index, composite variable ranks had a much higher concordance (Kendall's *W* = 0.42, P<0.0001), most likely owing to the correlation imposed by higher absolute consumption and economic activity in populous and wealthy (in absolute terms) countries (see also below).

### Correlation with existing environmental performance indicators

There was evidence of moderate correlation and concordance among the different composite indicators compared. Overall concordance among the five indicator ranks (our composite proportional index, EPI, HDI, GSI and EF) gave a Kendall's *W* = 0.25 (P = 0.04; *n* = 110 countries with data for all 5 indices; Fig. S1). EPI and HDI were positively correlated (Kendall's *τ* = 0.698, P<0.0001), and HDI and EF were negatively correlated (Kendall's *τ* = −0.670, P<0.0001). There was a weak negative relationship between our composite proportional environmental impact rank and EPI (Kendall's *τ* = −0.21, P = 0.0001, *n* = 149 countries), HDI (Kendall's *τ* = −0.22, P<0.0001, *n* = 178 countries), and GSI (Kendall's *τ* = −0.25, P<0.0001, *n* = 118 countries), but only the suggestion of a weak positive relationship between the proportional environmental impact rank and EF (Kendall's *τ* = 0.09, P = 0.0991, *n* = 150 countries; Fig. S2).

The relationships were much weaker or non-evident when considering the absolute environmental impact rank; there was no concordance among the five indicators (Kendall's *W* = 0.19, P = 0.69; *n* = 110 countries), and only EPI was negatively correlated with the absolute environmental impact rank (Kendall's *τ* = −0.12, P = 0.037, *n* = 149 countries).

### Correlation with socio-economic ranks

**Figure 2:**
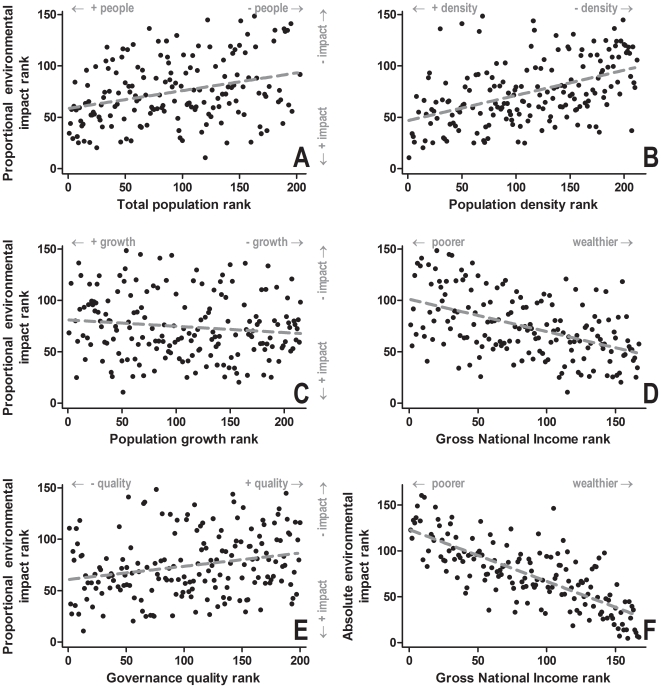
Bivariate correlations among environmental impact ranks and socio-economic variable ranks based on Kendall's *τ*. Strength of the relationships for which evidence exists between relative and absolute environmental impact ranks (see text for details) and socio-economic variables (human population size, human population density and human population growth rate, wealth [purchase power parity-adjusted Gross National Income] and governance quality) as measured by *τ* are given in the Results.

The composite proportional environmental impact rank correlated with the five socio-economic ranks (Fig. 2). We found that countries with higher total human populations and densities had greater proportional environmental impact (Kendall's *τ* = 0.209 and 0.336, P<0.0001, respectively), and those with lower population growth rates had a slightly lower proportional environmental impact (Kendall's *τ* = 0.114, P = 0.029) (Fig. 2A–C). Countries with greater total wealth (total purchasing power parity-adjusted Gross National Income) had worse environmental records than poorer countries (*τ* = −0.331, P<0.0001) (Fig. 2D), and those with poorer governance had slightly higher environmental impact; *τ* = 0.180, P = 0.0005) (Fig. 2E). However, none of the socio-economic ranks correlated with the absolute environmental impact rank except total purchasing power parity-adjusted Gross National Income (*τ* = −0.537, P<0.0001; Fig. 2F).

**Figure 3:**
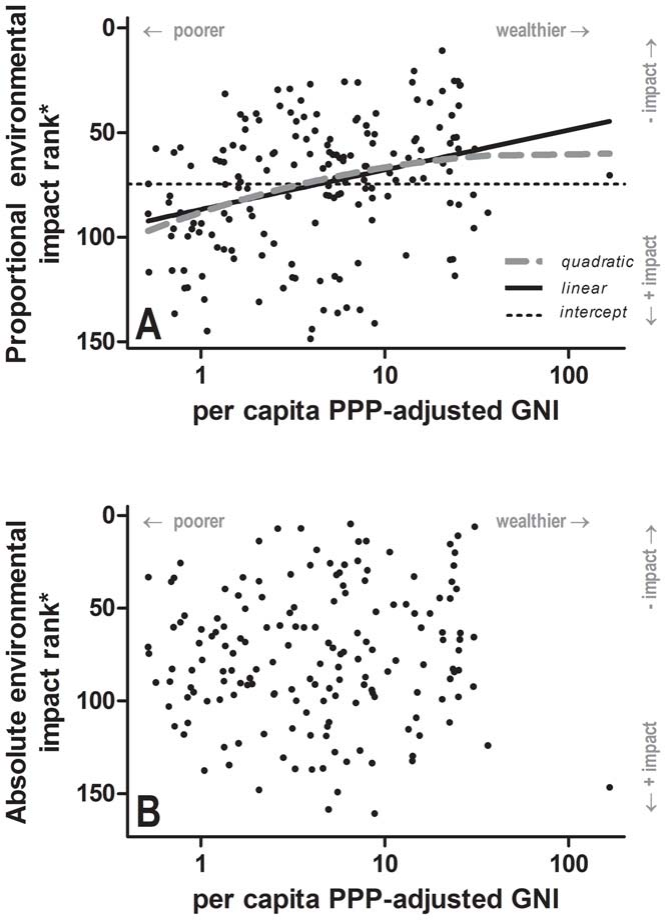
Tests for the environmental Kuznets curve (EKC) hypothesis. The EKC asserts that environmental impact is a non-linear function of per capita wealth [10]. Top panel: the intercept-only, linear, and quadratic (on log_10_ scale) models fitted to the proportional environmental impact rank. The linear model had the highest Bayesian inference support (see Results). Bottom panel: the intercept-only model (i.e., no relationship) had the highest support for the absolute environmental rank. The ‘*’ indicates an opposite rank direction to that presented in Fig. 2 for mathematical convenience (i.e., fitting a nonlinear function to the data).

Our choice to focus on absolute (rather than per capita) measures of wealth will tend to identify the largest economies as having the greatest environmental impacts. To test the EKC hypothesis explicitly, we found evidence for a positive relationship between the proportional environmental impact rank and per capita wealth; i.e., as per capita wealth increases, proportional environmental impact decreases (Fig. 3A). This was also supported by comparing the ranks using Kendall's *τ* (*τ* = −0.210, P<0.0001). The log-linear model (*w*BIC = 0.891) explained 9.6% of the deviance in the data and was 8.4 times more likely (BIC evidence ratio) than the quadratic model (*w*BIC = 0.107; Fig. 3A). Thus, there is little evidence for the EKC hypothesis, although there was a slight improvement in proportional environmental performance as per capita wealth increased. There was no relationship between the absolute environmental rank and per capita GNI-PPP (Fig. 3B).

**Figure 4:**
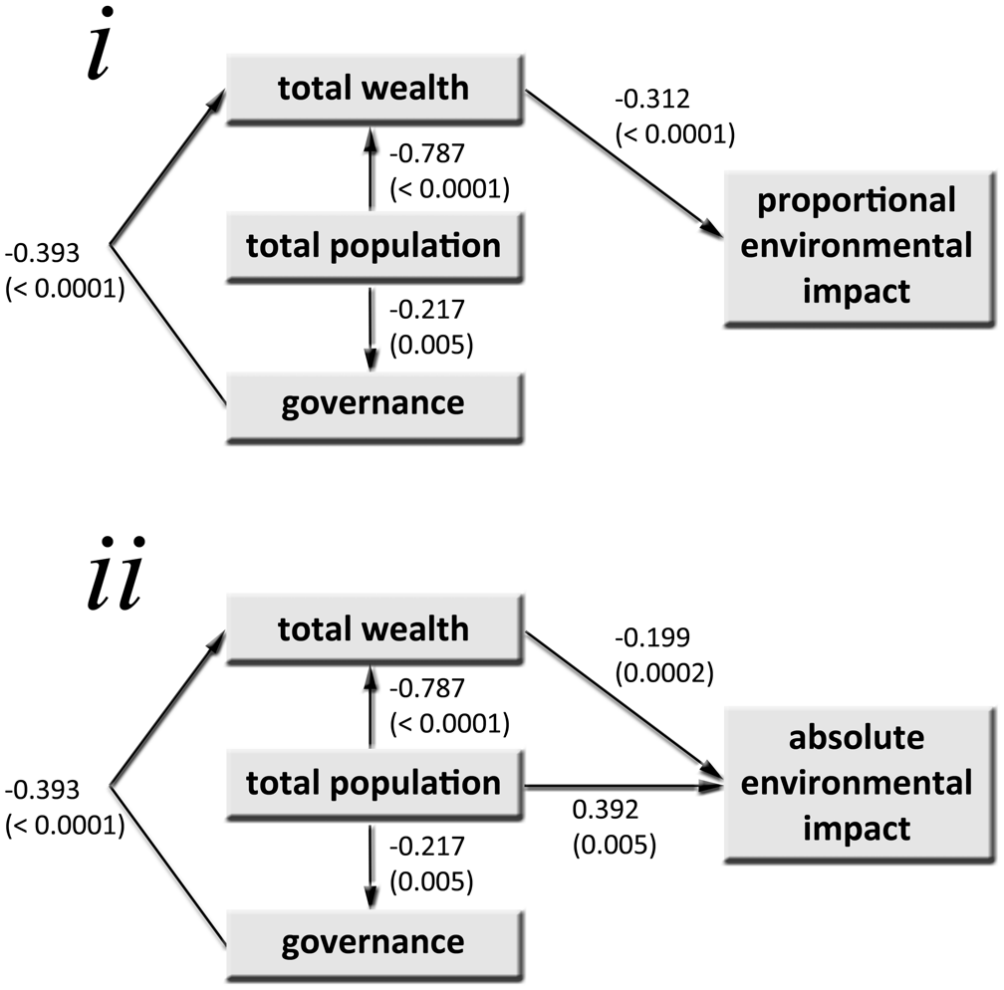
Structural equation models for environmental impact ranks. Top Bayesian Information Criterion- (BIC-) ranked structural equation models for the (*i*) proportional environmental impact rank (Model A; Table 4*i*) and (*ii*) absolute environmental impact rank (Model D; Table 4*ii*). Wealth (purchasing power parity-adjusted Gross National Income) had the highest correlation with proportional and absolute rank, with some additional contribution of total human population size to the absolute rank. Numbers shown are path coefficients with associated Type I error (P) probabilities. See full model rankings in Table 4.

Of course, identifying the causative aspect of these correlates is problematic because of the strong inter-correlation of predictor ranks (Table S9). As human population size increases, total wealth increases, and governance quality decreases. Likewise, there is a positive correlation between wealth and governance quality, such that poorer countries have lower-quality governance. Structural equation models (SEM) revealed total human wealth is the most important correlate of both relative and absolute environmental impact (Table 8), with lesser contributions from population size and governance quality (Fig. 4). Structural model ‘A’ that contains total wealth (GNI-PPP) as the only correlate of proportional environmental impact rank was the top-ranked SEM (*w*BIC = 0.439), but there was also some support for models D and E (*w*BIC = 0.291 and 0.268, respectively) (Fig. 4; Table 8). For absolute environmental impact, model ‘D’ including wealth and population size was the highest-ranked model (*w*BIC = 0.763; Table 8; Fig. 4). Model coefficients indicate that increasing total wealth is strongly correlated with higher proportional and absolute environmental impact, and increasing population size explains additional variance in absolute environmental impact (Fig. 4).

**Table 8:** Ranking of six candidate path models relating socio-economic variables to the (*i*) proportional (pENV) and (*ii*) absolute environmental impact rank (aENV) based on the Bayesian Information Criterion (BIC).

Model Index	Model	df	χ^2^	AGFI	BIC	ΔBIC	*w*BIC
(*i*) Proportional environmental impact rank							
A	pENV∼*gni*	2	4.803	0.930	−5.433	0.000	0.439
D	pENV∼*pop + gni*	1	0.508	0.985	−4.610	0.823	0.291
E	pENV∼*gni + gov*	1	0.673	0.980	−4.445	0.988	0.268
F	pENV∼*pop + gov*	1	10.453	0.704	5.335	10.768	0.002
B	pENV∼*pop*	2	32.521	0.592	22.285	27.718	<0.001
C	pENV∼*gov*	2	37.776	0.538	27.540	32.973	<0.001
(*ii*) Absolute environmental impact rank							
D	aENV∼*pop + gni*	1	0.669	0.980	−4.449	0.000	0.763
F	aENV∼*pop + gov*	1	3.114	0.908	−2.004	2.445	0.225
B	aENV∼*pop*	2	13.965	0.806	3.729	8.178	0.013
E	aENV∼*gni + gov*	1	34.207	0.148	29.089	33.538	<0.001
A	aENV∼*gni*	2	60.462	0.338	50.226	54.675	<0.001
C	aENV∼*gov*	2	203.537	−0.305	193.301	197.749	<0.001

(*i*) For pENV, models A, D and E have high adjusted goodness-of-fit index (AGFI). Model A is the most highly ranked relative to other models in the candidate set based on BIC weights (*w*BIC), with lower support for models D and E. All remaining models have little support. (*ii*) For aENV, only models D and F have high AGFI. Model D is the most highly ranked relative to other models in the candidate, and with less support for model F. All remaining models have little support. Shown also are the model degrees of freedom (df), associated model Chi-square value (χ^2^), and the difference between the top-ranked model's BIC and that of the model under consideration (ΔBIC). Model variables include environmental rank (pENV or aENV), total human population size rank (*pop*), purchasing power parity-adjusted Gross National Income (*gni*) rank and governance quality rank (*gov*) (see text for details). Each model is described by the hypothetical causal paths between socio-economic indicators and environmental impact. Refer to Fig. S4 for full path model details.

## Discussion

Our results based on a novel and objective combination of proportional and absolute environmental impact variables (as opposed to metrics that incorporate human health and/or economic indicators directly – see [8] for a review) demonstrate that overall wealth is the most important correlate of environmental impact, although population size explains additional variation in absolute impact. The modest concordance we found among common sustainability ranking systems is partially due to our choice to exclude economic and human health indicators, with our indices thus providing a more direct measure of environmental impact than of ‘sustainability’ (i.e., the capacity for ecosystems and human living standards to endure) *per se*. Indeed, many existing metrics of environmental impact attempt to make predictions of future resource use and so require imposing many untestable assumptions on their metrics (e.g., Ecological Footprint; [8]). Our ranking system explicitly avoids such assumptions and instead focuses on measures of present-day accumulated environmental degradation. Of course, the purpose of any index of environmental impact depends on its ultimate application – proportional impact is a better reflection of a country's performance relative to economic opportunity, irrespective of contextual wealth and population size. However, if one desires to measure a country's contribution to global environmental degradation, then absolute environmental impact is a better reflection of a country's contribution to the world's current environmental state.

We openly acknowledge that because our aim was to provide as parsimonious an environmental impact index as possible (maximizing sample sizes and data availability), we could not incorporate all major indices of environmental degradation. Measures such as the magnitude of bushmeat harvest [30], coral reef habitat quality [31], seagrass loss [32], freshwater habitat degradation [33], illegal fishing [34], invertebrate threat patterns [35], and some forms of greenhouse gas emission [36] were simply not available at the global scale of investigation. Nonetheless, we contend that our indices provide the most comprehensive measures of relative country-level environmental impact derived from the most complete global datasets available. The low sensitivity of each ranking to the exclusion of component metrics reinforces their robustness.

Despite the different derivation and application of proportional and absolute ranks, we found a surprising correlation between the two. This suggests that a country's consumption, pollution and land-use trends relative to opportunity reflect, at least to some degree, its citizens' attitude to environmental stewardship globally. The most striking aspect of this correlation was the dominance of Asian countries (Fig. 1) within the highest proportional and absolute rankings; China, Indonesia, Japan, Malaysia, Thailand, and Philippines had proportionally and absolutely the highest environmental impact according to our composite indices (Tables 2 & 3). Of course, our indices naturally focus on modern environmental impact (by their very construction we were limited to environmental impacts occurring within the last few decades); thus, they ignore some elements of historical degradation (e.g., deforestation in Europe). The corollary is that the proportional index in particular might penalize developing nations more heavily, even though some European nations still performed poorly (e.g., Denmark, Netherlands, Malta). Nonetheless, future policies developed using our index cannot address ancient environmental misconduct – they can only attempt to rectify current and future destructive practices.

Our composite index also revealed an interesting, perhaps paradoxical, result with respect to the predictions arising from the environmental Kuznets curve (EKC) hypothesis [10]. The EKC predicts that wealthier societies can reduce environmental degradation beyond a certain threshold [10]. Our explicit tests of non-linearity in the relationship between per capita wealth and environmental impact supported only linear (proportional impact) or no relationship (absolute impact; Fig. 3). Although the EKC prediction was not supported, we did find a weak correlation between per capita wealth and proportional environmental impact, suggesting that some gains in environmental performance can be achieved with increasing per capita wealth. However, our general finding that absolute wealth was the principal correlate of higher environmental impact suggests that any potential improvement resulting from higher per capita wealth is overwhelmed by the current necessity for economies to grow. We add though that temporally static (mean) measures of environmental performance compared across spatial gradients (countries) might obscure temporal patterns within countries. Therefore, evaluating temporal progress and the role of more environmentally friendly technologies and better education within a country might reveal that the EKC is still valid, at least under certain socio-economic circumstances and for particular measures of environmental performance. On the other hand, increasing trade liberation could make EKCs increasingly difficult to test because of externalities (import and export).

Governance quality has been linked to environmental degradation [23], [37]; however, our analyses revealed that it was the least important of the three plausible drivers of environmental impact among countries. We hypothesize that this arises because better governance drives economic development, urbanization, habitat loss and the resultant environmental impact (see Table S9 for correlations). Conversely, countries with poor governance and political corruption might experience a high deforestation rate owing to poor forestry practices and illegal logging [20], [22], [23], and consequently, high species endangerment. The lack of a strong effect might also arise from changing governance quality over time that is not necessarily reflected in average ranks.

Our rankings are not meant to excuse better-ranked countries from their environmental responsibilities; rather, the correlations identified suggest that there are several policies that can assist in reducing overall environmental impact. Human population size and wealth are intrinsically linked, meaning that one will most likely change in response to changes in the other, regardless of setting. Richer countries generally exploit more resources for the same population size as the relationship between human population (total and density) and proportional environmental impact suggests, but as per capita resource availability declines, environmental impact increases. A fundamental tenet of population ecology is that per capita resources decline as populations near carrying capacity, so the absolute pressure on the environment is dictated more by variation in a country's ‘carrying capacity’ than absolute population size or per capita resources use.

This assumes though that carrying capacity is not modified via ‘leakage’, that is, externalizing environmental damage via pollution trading and outsourcing environmentally intensive production processes [38]. In a more modern context, leakage might be substantial when measured via carbon outputs from highly industrialized countries; without full greenhouse gas accounts available for each country, environmental quality per nation cannot be linked as directly to within-nation policies and behaviors. For example, Costa Rica's recent reduction in deforestation rate appears to have been offset by increasing timber imports from elsewhere [39], and Japan's maintenance of its forest is supported by extensive timber imports from South East Asia and beyond [40]. Although we could not consider the leakage effect directly given that there are few global-scale datasets available [38], [39], we expect that leakage is either proportional to absolute wealth and intensity of development, or it could even increase (e.g., exponentially) with increasing wealth. If leakage is an important phenomenon at the global scale, the importance of increasing wealth on environmental degradation would be even greater than we identified here. The existence of substantial leakage also erodes support for the EKC hypothesis [38].

Our results show that the global community not only has to encourage better environmental performance in less-developed countries, especially those in Asia, there is also a requirement to focus on the development of environmentally friendly practices in wealthier countries. However, populous countries currently undergoing rapid economic development such as China [41], India and Indonesia might have the fastest increases in environmental impact and are thus the regions where improved environmental protection policies stand to benefit the most people. Improving policy and practice in these regions might also provide a larger global benefit, because the beneficiaries of the policies that our ranking system could influence would often be global in extent (e.g., international trade treaties, carbon tax, development aid). However, we recommend that policy makers avoid using our metrics to prioritize biodiversity conservation spending explicitly because usually finer-scale cost-benefit analyses are required to maximize the number of species protected per monetary unit spent [42]. While some aspects of environmental impact considered are generally irreversible in the short term (e.g., forest loss and species endangerment), others can be reversed by institutionalizing sustainable development policies that limit consumption [1].

## Materials and Methods

### Environment

The following variables were combined (see *Analysis*) to produce relative and absolute ranks of a country's environmental impact. We were unable to include variables such as bushmeat extraction, coral reef quality, seagrass change, and freshwater habitat loss given a lack of country-specific data (see Discussion).

#### Natural forest loss

We obtained plantation forest area and total forest area from 1990 and 2005 from the Food and Agriculture Organization (FAO) Global Forest Resources Assessment 2005 (www.fao.org). Area of natural forest of each country was calculated by subtracting plantation forest area from total forest area. Absolute natural forest area change was defined as the difference in natural forest area between years 1990 and 2005. For the proportional index, this difference was converted to a proportion of total country area to standardize among countries.

#### Natural habitat conversion

We evaluated the degree of historical habitat loss by overlaying a modified version of the Global Land Cover 2000 dataset [43], [44] over a map of global political boundaries [45] in ArcGIS v. 9.2. For the proportional index, we calculated historical habitat loss by expressing the area of human-modified land-cover as a proportion of total terrestrial land area in each ecoregion. Our definition of human-modified land-cover included cultivated and managed land, cropland mosaics, and artificial surfaces and associated areas.

#### Marine captures

We compiled marine capture data using the FAO FISHSTAT Plus Ver. 2.32 software [46]. Volume of marine captures by each country was collated from the Capture Production 1950–2006 dataset (ftp://ftp.fao.org). Fisheries exploitation was assessed by computing the 10-year average total capture of marine fish, whales, seals and walruses. For the proportional index, marine captures were standardized among countries by expressing the values as a proportion of the total coastline distance (km) for countries possessing a marine Exclusive Economic Zone (sourced from www.earthtrends.wri.org). For countries with no marine captures or coastline, proportions were set to zero. Although illegal, unreported and unregulated (IUU) fishing is estimated to comprise a large component of marine captures worldwide [34], but its very nature, few country-specific data exist [47]. Therefore, we could not incorporate this additional measure.

#### Fertilizer use

The excessive application of nitrogen, phosphorous and potassium (NPK) fertilizers can result in the leaching of these chemicals into water bodies and remove, alter or destroy natural habitats [47], [48]. The consumption of nitrogen (N), phosphorous (P_2_O_5_) and potassium fertilizers (K_2_O) by each country from years 2002 to 2005 was compiled from the FAOSTAT database (www.faostat.fao.org). The countries were ranked by the average annual consumption of all three categories of fertilizers. For the proportional index, we calculated the average NPK fertilizer consumption per unit area of arable land (100 g/ha of arable land) [49].

#### Water pollution

We obtained data describing the total yearly (1995–2004) emissions of organic water pollutants measured by biochemical oxygen demand (BOD); this index describes the amount of oxygen consumed by bacteria decomposing waste in water (kg/day) [49]. For the proportional index, we divided the BOD values by the maximum theoretical yearly amount of water available for a country at a given moment (Total Actual Renewable Water Resources obtained from the FAO AQUASTAT database [50]. We took the mean of the Total Actual Renewable Water Resources data from three time slices (1993–1997, 1998–2002, 2003–2007) which correspond to the periods covered by the BOD data.

#### Carbon emissions

The two main anthropogenically driven sources of atmospheric CO_2_ driving rapid climate change [51] are large-scale burning of fossil fuels for energy and the clearing of forest and woodlands [52]. CO_2_ emissions data from the flaring of natural gas and burning of fossil fuels were compiled from the Energy Information Administration (EIA) database (www.eia.doe.gov). Countries were ranked by computing the most recent 10-year average (years 1996–2005) CO_2_ emissions.

CO_2_ emissions from land-use change and forestry were compiled using the Climate Analysis Indicators Tool (CAIT) (http://cait.wri.org). These estimates were based on a global and regional analysis of land-use change [53]. The types of land-use change and forestry activities assessed included 1. clearing of natural ecosystems for permanent croplands, 2. clearing of natural ecosystems for permanent pastures, 3. abandonment of croplands and pastures with subsequent recovery of carbon stocks to those of the original ecosystem, 4. shifting cultivation, and 5. industrial and fuel wood harvest (emissions of carbon from wood products included) (http://cait.wri.org). We could not include emissions from shipping and flights because the data are not yet incorporated into country-specific accounting methods. Data collected were the most recent 10-year average (years 1991–2000) CO_2_ emissions from land-use change and forestry datasets. Total CO_2_ emissions were the sum of fossil fuel and land-use means, and these were standardized among countries for the proportional index by dividing by total country area.

#### Biodiversity threat

To quantify the threat to biodiversity in each country, we chose for our analyses the three best-documented animal taxa (i.e., birds, amphibians, and terrestrial mammals) assessed using standardized IUCN Red List (www.iucnredlist.org) criteria. These three taxa have either been completely assessed (birds, amphibians) or almost completely assessed (mammals) [54]. BirdLife International, the Red List Authority for birds, has assessed all 10104 bird species in its 2008 Red List [55]. Similarly, 6260 amphibian species have been evaluated by the IUCN (www.iucnredlist.org/amphibians/). As for the mammals, all species listed in [56] were assessed in the 1996 Red List. Some species were re-evaluated and newly-described species evaluated in subsequent editions of the Red List. However, owing to taxonomic changes in existing mammal species and discoveries of new species, assessment gaps still exist (www.iucnredlist.org). Despite this, because mammals represent one of the most important groups of species in terms of evolution, ecology and economic impact (www.iucnredlist.org), we believe that the inclusion of terrestrial mammals in our analyses is merited. Threat status of birds native to each country was compiled from the World Bird Database (www.birdlife.org). Threat status of amphibians native to each country was compiled from the IUCN Amphibian database (www.iucnredlist.org/amphibians/). Threat status of mammals native to each country was compiled from the 2008 IUCN Red List (www.iucnredlist.org). For the proportional index, the number of threatened species was divided by the total number of species listed in the 2008 IUCN Red List. It is logical to posit that species endemic to a large country are less likely to be threatened because their potential range size is larger than that of species endemic to smaller countries. However, Giam et al. [57] found little evidence for an effect of country area on endemic plant threat risk, so the potential bias owing to country area is likely absent or weak.

### Socio-economic variables

We collected the following socio-economic variables summarized per country to examine their relationship with environmental impact ranks.

#### Human population size and growth

Total population size of each country was collated from the series ‘Population total (UN Population Division's annual estimates and projections)’ in the United Nations Common Database (http://unstats.un.org). Annual population figures were recorded for the period 1990–2005 (Fig. S3, top panel). These figures were used to derive the annual rate of population change, 15-year average population, and population density for each country.

#### Wealth

The purchasing power parity-adjusted gross national income (GNI-PPP) of each country for the period 1990–2005 was collated from the World Resources Institute (WRI) EarthTrends database (www.earthtrends.wri.org) (Fig. S3, middle panel).

#### Governance quality

Governance quality for each country was obtained from the Worldwide Governance Indicators (WGI) project [58], a metric that is strongly correlated with the better-known Corruption Perception Index (CPI; www.transparency.org) (mean 1996–2005 CPI versus the 2002–2006 World Bank governance indicator; Kendall's *τ* = 0.755; P<0.0001). We used the former metric because the WGI project appraised countries using indicators of six dimensions of governance: 1. voice and accountability, 2. political stability, 3. government effectiveness, 4. regulatory quality, 5. rule of law, and 6. control of corruption. For each of the six indicators, a score from −2.5 (lowest quality) to 2.5 (highest quality) was allocated to each country. We calculated average values of each of these six dimensions for each country from (2002–2006) to obtain a reasonable estimate of governance quality of each country. Factor analysis (not shown) extracted only one component consisting of all six dimensions revealing strong inter-correlations. We therefore reduced the six indicators into a single principal component that explained 87.7% of the total variance (Fig. S3, bottom panel).

### Analysis

#### Composite scores and ranks of variables

Statistical problems of autocorrelation render classic interpretations of socio-economic drivers of deforestation problematic [15], and conclusions vary depending on the technique used [8], [28]. To avoid temporal autocorrelation [15], we took temporal means over the periods indicated above for each environmental (or population) metric for both proportional and absolute composite ranks. For each environmental impact, human population density, human population growth rate, governance quality and wealth (purchasing power parity-adjusted Gross National Income) variable, we made simple hierarchical rankings (i.e., we did not consider the magnitude of the values' difference between countries; however, geometric mean rankings presented provide a measure of relative distance between countries in the final composite rank). Instead of averaging raw ranks for composite indices (environmental impact and human population pressure), we took the back-transformed mean of the log_10_-transformed rank value to avoid the undue influence of outliers (analogous to a geometric mean) [8]:
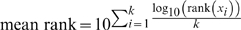
where *x_i_* = environmental metric *i* (for *k* metrics considered). For human population growth, we considered the back-transformed mean of the log_10_-transformed ranks derived from population density and population growth. For the environmental impact ranks (absolute and proportional), countries were removed if three or more indices contained no data; the final ranking was reasonably insensitive to the choice of the number of missing values allowed (Tables S1, S2, S3).

#### Correlations with other ranking indices

To determine the degree of concordance between the composite environmental degradation rank and other global indicators, we examined the relationship between our ranking and that derived from four other indicators covering a broad range of countries: the Environmental Performance Index (EPI), the Human Development Index (HDI), the Genuine Savings Index (GSI) and the Environmental Footprint (EF) index (other indices were excluded due to poor global coverage). General descriptions of the EPI, HDI, GSI and EF are provided in File S2. Ranks were compiled for each indicator and compared using Kendall's *τ* and concordance tests as described above.

#### Correlations with socio-economic variables

We examined bivariate correlations among ranks using Kendall's *τ* for ranked data, and concordance among composite variables was assessed using Kendall's *W*. We tested the environmental Kuznets curve (EKC) hypothesis [10] directly by contrasting three models to the proportional rank versus the log_10_-transformed per capita purchasing power parity-adjusted GNI: (1) the intercept-only model, (2) a log-linear model (i.e., linear on the log_10_ scale) and (3) a log-quadratic model. Evidence for the log-quadratic model would support the EKC hypothesis. We could not apply non-linear models to the fully ranked data; hence, it was necessary to compare the geometric mean proportional ranks and the raw wealth data (we used the log_10_ scale due to highly right-skewed per capita GNI-PPP). The Bayesian information criterion (BIC) was used to assign relative strengths of evidence to the different candidate models. The relative likelihoods of candidate models were calculated using BIC weights [59], [60], with the weight (*w*BIC) of any particular model varying from 0 (no support) to 1 (complete support) relative to the entire model set.

Identifying the causative aspect of socio-economic correlates is problematic because, while socioeconomic variables are correlated with environmental degradation, some are also inter-correlated (see Table S9). To overcome this problem, we used structural equation models (SEM) that involve partitioning simple correlations among a set of variables according to each hypothesized causal link (also commonly known as ‘path’ models) to test the descriptive ability of different models [61]. First, we built six candidate path models based on logic and previous studies to examine the socio-economic drivers of environmental impact (see also Fig. S4). The relationship between socio-economic variables is kept constant in all six models that can consider a maximum of two contributing correlates to environmental impact rank. Total human population (used instead of population density or growth rate because neither density nor growth rate is correlated with total population rank) is correlated with total wealth (Table S9), but imperfectly, so the inclusion of this path reveals whether population has any additional explanatory power after taking wealth into account. We also hypothesized that high total human population drives ineffective governance because high populations place a strain on governmental resources, thus increasing a country's susceptibility to low governance quality. Lastly, we hypothesized that good governance is a driver of higher wealth [62].

We fitted the six candidate path models (Fig. S4) to the data using the sem function implemented in *R* 2.7.2 [63]. We used BIC weights to assign relative strength of evidence to the candidate models. The goodness-of-fit of the candidate models to the data was evaluated using the adjusted goodness-of-fit statistic provided by the sem function.

## Supporting Information

File S1 (0.07 MB RTF)

File S2 (0.08 MB RTF)

Table S1 Twenty worst-ranked countries by proportional composite environmental (pENV) rank (lower ranks = higher negative impact) when only two environmental variables were allowed to be missing (cf. three missing for rankings in main text and four missing in Table S2). Shown are country names and codes, population density (PD) rank, population growth rate (PGR) rank, governance quality (GOV) rank, Gross National Income (GNI) rank, natural forest loss (NFL) rank, natural habitat conversion (HBC) rank, marine captures (MC) rank, fertilizer use (FER) rank, water pollution (WTP) rank, proportion of threatened species (PTHR) rank, and carbon emissions (CO2) rank. Constituent variables used to create the pENV are in boldface. See text for details. Missing values denoted by ‘-’.
(0.17 MB RTF)

Table S2 Twenty worst-ranked countries by proportional composite environmental (pENV) rank (lower ranks = higher negative impact) when four environmental variables were allowed to be missing (cf. three missing for rankings in main text and two missing in Table S1). Shown are country names and codes, population density (PD) rank, population growth rate (PGR) rank, governance quality (GOV) rank, Gross National Income (GNI) rank, natural forest loss (NFL) rank, natural habitat conversion (HBC) rank, marine captures (MC) rank, fertilizer use (FER) rank, water pollution (WTP) rank, proportion of threatened species (PTHR) rank, and carbon emissions (CO2) rank. Constituent variables used to create the pENV are in boldface. See text for details. Missing values denoted by ‘-’.
(0.17 MB RTF)

Table S3 Twenty top-ranked countries by proportional composite environmental (pENV) rank (higher ranks = lower negative impact) when only two environmental variables were allowed to be missing (cf. three missing for rankings in main text and four missing in Table S4). Shown are country names and codes, population density (PD) rank, population growth rate (PGR) rank, governance quality (GOV) rank, Gross National Income (GNI) rank, natural forest loss (NFL) rank, natural habitat conversion (HBC) rank, marine captures (MC) rank, fertilizer use (FER) rank, water pollution (WTP) rank, proportion of threatened species (PTHR) rank, and carbon emissions (CO2) rank. Constituent variables used to create the pENV are in boldface. See text for details. Missing values denoted by ‘-’.
(0.17 MB RTF)

Table S4 Twenty top-ranked countries by proportional composite environmental (pENV) rank (higher ranks = lower negative impact) when four environmental variables were allowed to be missing (cf. three missing for rankings in main text and two missing in Table S3). Shown are country names and codes, population density (PD) rank, population growth rate (PGR) rank, governance quality (GOV) rank, Gross National Income (GNI) rank, natural forest loss (NFL) rank, natural habitat conversion (HBC) rank, marine captures (MC) rank, fertilizer use (FER) rank, water pollution (WTP) rank, proportion of threatened species (PTHR) rank, and carbon emissions (CO2) rank. Constituent variables used to create the pENV are in boldface. See text for details. Missing values denoted by ‘-’.
(0.17 MB RTF)

Table S5 List of 49 missing countries from the proportional environmental impact ranking; minimum criterion for inclusion was ≤3 missing environmental variable values.
(0.09 MB RTF)

Table S6 Full list of 179 countries ranked by proportional composite environmental (pENV) rank (lower ranks = higher negative impact). Shown are country names and codes, population density (PD) rank, population growth rate (PGR) rank, governance quality (GOV) rank, Gross National Income (GNI) rank, natural forest loss (NFL) rank, natural habitat conversion (HBC) rank, marine captures (MC) rank, fertilizer use (FER) rank, water pollution (WTP) rank, proportion of threatened species (PTHR) rank, and carbon emissions (CO2) rank. Constituent variables used to create the pENV are shaded. See text for details. Missing values denoted by ‘-’.
(0.83 MB RTF)

Table S7 List of 57 missing countries from the absolute environmental impact ranking; minimum criterion for inclusion was ≤3 missing environmental variable values.
(0.10 MB RTF)

Table S8 Full list of 171 countries ranked by absolute composite environmental (aENV) rank (lower ranks = higher negative impact). Shown are country names and codes, population density (PD) rank, population growth rate (PGR) rank, governance quality (GOV) rank, Gross National Income (GNI) rank, natural forest loss (NFL) rank, natural habitat conversion (HBC) rank, marine captures (MC) rank, fertilizer use (FER) rank, water pollution (WTP) rank, proportion of threatened species (PTHR) rank, and carbon emissions (CO2) rank. Constituent variables used to create the aENV are shaded. See text for details. Missing values denoted by ‘-’.
(0.82 MB RTF)

Table S9 Kendall's rank correlation (*τ*) matrix for socio-economic ranks: POP = human population size (2005), POPD = human population density (2005), PGR = human population growth rate (1990–2005), GNI = purchasing power parity-adjusted Gross National Income, GOV = governance quality. Lower-left quadrant values are Kendall's *τ*; upper-right quadrant values are Type I error probabilities for the coefficients. Boldface *τ* indicate sufficient evidence of a relationship.
(0.09 MB RTF)

Figure S1 Rank correlations between existing environmental indicators. EPI = Environmental Performance Index, HDI = Human Development Index, GSI = Genuine Savings Index, EF = Ecological Footprint [reviewed in 8].
(0.83 MB TIF)

Figure S2 Rank correlations between proportional environmental impact (pENV) ranks and four existing environmental indicator ranks. EPI = Environmental Performance Index, HDI = Human Development Index, GSI = Genuine Savings Index, EF = Ecological Footprint [reviewed in 8].
(0.86 MB TIF)

Figure S3 World distribution of socio-economic variables. Relative distributions of global human population (2005) (top panel: dark red = highest population), wealth rank (middle panel: dark blue = wealthiest based on purchasing power parity-adjusted Gross National Income) and governance quality rank (bottom panel: dark green = highest quality) among countries.
(1.44 MB TIF)

Figure S4 Path diagrams for the seven competing structural equation models A to G. Single-headed arrows represent hypothesized direct effects of one variable on another.
(1.90 MB TIF)

## References

[1] Ehrlich PR, Pringle RM (2008). Where does biodiversity go from here? A grim business-as-usual forecast and a hopeful portfolio of partial solutions.. Proceedings of the National Academy of Sciences of the USA.

[2] Bradshaw CJA, Sodhi NS, Brook BW (2009). Tropical turmoil – a biodiversity tragedy in progress.. Frontiers in Ecology and the Environment.

[3] Steffen W, Crutzen PJ, McNeill JR (2007). The Anthropocene: are humans now overwhelming the great forces of nature?. Ambio.

[4] Daily GC (1997). Nature's Services.

[5] Millennium Ecosystem Assessment (2005). Ecosystems and Human Well-being: Synthesis.

[6] U.S. Census Bureau (2008). International Data Base (IDB).

[7] United Nations (2004). Issues Paper for the Session on Natural Resource Governance and Conflict Prevention, Expert Group Meeting on Conflict Prevention, Peacebuilding and Development.

[8] Böhringer C, Jochem PEP (2007). Measuring the immeasurable - a survey of sustainability indices.. Ecological Economics.

[9] Strassburg BBN, Kelly A, Balmford A, Davies RG, Gibbs HK (2010). Global congruence of carbon storage and biodiversity in terrestrial ecosystems.. Conservation Letters.

[10] Stern DI, Common MS, Barbier EB (1996). Economic growth and environmental degradation: the environmental Kuznets curve and sustainable development.. World Development.

[11] Clausen R, York R (2008). Global biodiversity decline of marine and freshwater fish: a cross-national analysis of economic, demographic, and ecological influences.. Social Science Research.

[12] Shi A (2003). The impact of population pressure on global carbon dioxide emission, 1975–1996: evidence from pooled cross-country data.. Ecological Economics.

[13] Foster JB (1992). The absolute general law of environmental degradation under capitalism.. Capitalism, Nature, Socialism.

[14] York R, Rosa EA, Dietz T (2003). Footprints on the Earth: the environmental consequences of modernity.. American Sociological Review.

[15] Scrieciu SS (2007). Can economic causes of tropical deforestation be identified at a global level?. Ecological Economics.

[16] Barbier EB, Burgess JC (2001). The economics of tropical deforestation.. Journal of Economic Surveys.

[17] Naidoo R, Adamowicz WL (2001). Effects of economic prosperity on numbers of threatened species.. Conservation Biology.

[18] Hoffmann JP (2004). Social and environmental influences on endangered species: a cross-national study.. Sociological Perspectives.

[19] Czech B, Krausman PR, Devers PK (2000). Economic associations among causes of species endangerment in the United States.. BioScience.

[20] Ewers RM (2006). Interaction effects between economic development and forest cover determine deforestation rates.. Global Environmental Change.

[21] Didia DO (1997). Democracy, political instability and tropical deforestation.. Global Environmental Change.

[22] Geist HJ, Lambin EF (2002). Proximate causes and underlying driving forces of tropical deforestation.. BioScience.

[23] Smith RJ, Muir RDJ, Walpole MJ, Balmford A, Leader-Williams N (2003). Governance and the loss of biodiversity.. Nature.

[24] Transparency International (2002). Corruption Perception Index 2002.

[25] Li Q, Reuveny R (2006). Democracy and environmental degradation.. International Studies Quarterly.

[26] Jepson P, Jarvie JK, MacKinnon K, Monk KA (2001). The end for Indonesia's lowland forests?. Science.

[27] Morse S (2006). Is corruption bad for environmental sustainability? a cross-national analysis.. Ecology and Society.

[28] Bawa KS, Dayanandan S (1997). Socioeconomic factors and tropical deforestation.. Nature.

[29] Chape S, Harrison J, Spalding M, Lysenko I (2005). Measuring the extent and effectiveness of protected areas as an indicator for meeting global biodiversity targets.. Philosophical Transactions of the Royal Society B.

[30] Milner-Gulland EJ, Bennett EL (2003). Wild meat: the bigger picture.. Trends in Ecology and Evolution.

[31] Bellwood DR, Hughes TP, Folke C, Nystrom M (2004). Confronting the coral reef crisis.. Nature.

[32] Orth RJ, Carruthers TJB, Dennison WC, Duarte CM, Fourqurean JW (2006). A global crisis for seagrass ecosystems.. BioScience.

[33] Dudgeon D, Arthington AH, Gessner MO, Kawabata ZI, Knowler DJ (2006). Freshwater biodiversity: importance, threats, status and conservation challenges.. Biological Reviews.

[34] Agnew DJ, Pearce J, Pramod G, Peatman T, Watson R (2009). Estimating the worldwide extent of illegal fishing.. PLoS One.

[35] Butchart SHM, Stattersfield AJ, Baillie J, Bennun LA, Stuart SN (2005). Using Red List Indices to measure progress towards the 2010 target and beyond.. Philosophical Transactions of the Royal Society B.

[36] Olsthoorn X (2001). Carbon dioxide emissions from international aviation: 1950–2050.. Journal of Air Transport Management.

[37] Laurance WF (2007). Forest destruction in tropical Asia.. Current Science.

[38] Ghertner DA, Fripp M (2007). Trading away damage: quantifying environmental leakage through consumption-based, life-cycle analysis.. Ecological Economics.

[39] Vöhringer F (2004). Forest conservation and the clean development mechanism: lessons from the Costa Rican protected areas project.. Mitigation and Adaptation Strategies for Global Change.

[40] Kauppi PE, Ausubel JH, Fang J, Mather AS, Sedjo RA (2006). Returning forests analyzed with the forest identity.. Proceedings of the National Academy of Sciences of the USA.

[41] Guan D, Peters GP, Weber CL, Hubacek K (2009). Journey to world top emitter: an analysis of the driving forces of China's recent CO_2_ emissions surge.. Geophysical Research Letters.

[42] Wilson KA, McBride MF, Bode M, Possingham HP (2006). Prioritizing global conservation efforts.. Nature.

[43] Hoekstra JM, Boucher TM, Ricketts TH, Roberts C (2005). Confronting a biome crisis: global disparities of habitat loss and protection.. Ecology Letters.

[44] European Commission's Joint Research Centre (2002). GLC 2000: Global land cover mapping for the year 2000.. Institute for Environment and Sustainability.

[45] Hijmans R, Garcia N, Kapoor J, Rala A, Maunahan A (2008). Global Administrative Areas Version 0.9.. University of California at Berkeley.

[46] Food and Agriculture Organization (2007). FISHSTAT Plus: Universal Software for Fishery Statistical Time Series. Version 2.32.

[47] Halpern BS, Walbridge S, Selkoe KA, Kappel CV, Micheli F (2008). A global map of human impact on marine ecosystems.. Science.

[48] Vitousek PM, Aber JD, Howarth RW, Likens GE, Matson PA (1997). Human alteration of the global nitrogen cycle: sources and consequences.. Ecological Applications.

[49] The World Bank (2008). World Development Indicators 2008.

[50] Food and Agriculture Organization (2009). AQUASTAT: Global information system on water and agriculture.

[51] Oreskes N (2004). Beyond the ivory tower: the scientific consensus on climate change.. Science.

[52] Malhi Y, Grace J (2000). Tropical forests and atmospheric carbon dioxide.. Trends in Ecology and Evolution.

[53] Houghton RA (2003). Revised estimates of the annual net flux of carbon to the atmosphere from changes in land use and land management 1850–2000.. Tellus.

[54] Schipper J, Chanson JS, Chiozza F, Cox NA, Hoffmann M (2008). The status of the world's land and marine mammals: diversity, threat, and knowledge.. Science.

[55] BirdLife International (2008). The BirdLife Checklist of the Birds of the World, with Conservation Status and Taxonomic sources.. Version.

[56] Wilson DE, Reeder DM (2005). Mammal Species of the World: A Taxonomic and Geographic Reference.

[57] Giam X, Bradshaw CJA, Tan HTW, Sodhi NS (2010). Future habitat loss and the conservation of plant biodiversity.. Biological Conservation.

[58] Kaufmann D, Kraay A, Mastruzzi M (2007). Governance Matters VI: Aggregate and Individual Governance Indicators, 1996–2006.

[59] Burnham KP, Anderson DR (2002). Model Selection and Multimodel Inference: A Practical Information-Theoretic Approach.

[60] Link WA, Barker RJ (2006). Model weights and the foundations of multimodel inference.. Ecology.

[61] Mitchell RJ (1992). Testing evolutionary and ecological hypotheses using path analysis and structural equation modelling.. Functional Ecology.

[62] Abed GT, Gupta S (2002). Governance, Corruption and Economic Performance.

[63] Fox J (2006). Structural Equation Modelling with the sem package in R.. Structural Equation Modelling.

