# Supplementary material for: Evaluating the Relative Environmental Impact of Countries
Source: PLoS One. 2010 May 3;5(5):e10440. doi: 10.1371/journal.pone.0010440 (PMC2862718; doi:10.1371/journal.pone.0010440)
Supplement: File S1 — (0.07 MB RTF) [file pone.0010440.s001.rtf]

File S1. Sensitivity analysis for the choice of maximum number of missing values
The number of missing environmental variables allowed to create the composite environmental impact rank was set arbitrarily at 3 (out of 7 variables total). To examine how this affected the relative rankings, we recalculated the environmental impact rank using a minimum of 2 (Table S1) and 4 (Table S2) missing variable thresholds. With the more conservative cut-off of 2 maximum missing variable values, the total number of countries considered fell from 179 to 166. The relative ranking was unchanged for the 20 worst-ranked countries (Table S1), except that Qatar was removed from the ranking. For the least-conservative scenario considered with a maximum of 4 missing values allowed, the number of countries considered increased from 179 to 193. There was no change in ranking from considering 3 (main text results) or 4 (Table S1) in the 20 worst-ranked countries.
For the best-ranked countries, the choice of minimum number of missing values had more influence on the ranking and composition of the countries appearing in the top 20 (i.e., least environmental impact); however, there was still good consistency. Of the 20 best-ranked countries using three missing values as a threshold (Table 2), 14 were also in the 20 best-ranking when only two missing variables were allowed (albeit their order was slightly altered; Table S3). When compared to the ranking resulting from allowing 4 variables to have missing values, the majority of the countries were still in the top 20 (13/20) (Table S4).
