# Supplementary material for: Evaluating the Relative Environmental Impact of Countries
Source: PLoS One. 2010 May 3;5(5):e10440. doi: 10.1371/journal.pone.0010440 (PMC2862718; doi:10.1371/journal.pone.0010440)
Supplement: File S2 — (0.08 MB RTF) [file pone.0010440.s002.rtf]

File S2. Description of global indicators of environmental sustainability

The EPI [1] incorporates both environmental performance and human health data into a weighted, composite index to rank countries for environmental protection and 'sustainability'; the HDI [2] combines normalized measures of life expectancy, literacy, educational attainment and per capita gross domestic product (GDP); the GSI (also known as adjusted net saving) [3] measures the true rate of savings in an economy after taking into account investments in human capital, depletion of natural resources and damage caused by pollution; the EF [4] measures “the amount of biologically productive land and water area required to produce all the resources an individual, population, or activity consumes, and to absorb the waste they generate, given prevailing technology and resource management practices” [4].

References
1. Esty DC, Levy MA, Kim CH, de Sherbinin A, Srebotnjak T, et al. (2008) 2008 Environmental Performance Index. New Haven, Connecticut: Yale Center for Environmental Law and Policy.
2. Watkins K (2007) Human Development Report 2007/2008. New York: United Nations Development Programme, Palgrave Macmillan.
3. The World Bank (2006) Adjusted Net Saving. Washington, D.C.: The World Bank.
4. Ewing B, Goldfinger S, Wackernagel M, Stechbart M, Rizk S, et al. (2008) The Ecological Footprint Atlas 2008. Oakland, California: Global Footprint Network.
