# Supplementary material for: Evaluating the Relative Environmental Impact of Countries
Source: PLoS One. 2010 May 3;5(5):e10440. doi: 10.1371/journal.pone.0010440 (PMC2862718; doi:10.1371/journal.pone.0010440)
Supplement: Table S1 — Twenty worst-ranked countries by proportional composite environmental (pENV) rank (lower ranks = higher negative impact) when only two environmental variables were allowed to be missing (cf. three missing for rankings in main text and four missing in Table S2). Shown are country names and codes, population density (PD) rank, population growth rate (PGR) rank, governance quality (GOV) rank, Gross National Income (GNI) rank, natural forest loss (NFL) rank, natural habitat conversion (HBC) rank, marine captures (MC) rank, fertilizer use (FER) rank, water pollution (WTP) rank, proportion of threatened species (PTHR) rank, and carbon emissions (CO2) rank. Constituent variables used to create the pENV are in boldface. See text for details. Missing values denoted by ‘-’. (0.17 MB RTF) [file pone.0010440.s003.rtf]

Rank	Country	Code	PD	PGR	GOV	GNI	NFL	HBC	MC	FER	WTP	PTHR	CO2	pENV	
1	Singapore	SGP	1	51	13	115	128	5	91	1	4	63	1	10.6	
2	Rep Korea	KOR	14	158	56	154	23	61	20	17	21	29	5	20.4	
3	Kuwait	KWT	61	110	74	109	128	197	114	11	1	-	8	25.1	
4	Japan	JPN	23	188	30	165	87	87	18	21	29	13	6	25.2	
5	Thailand	THA	71	145	90	148	43	8	7	67	-	37	46	25.5	
6	Bahrain	BHR	6	41	73	52	-	193	59	4	-	123	2	25.7	
7	Malaysia	MYS	102	60	71	131	47	75	22	8	77	15	11	25.9	
8	Philippines	PHL	36	70	122	144	22	20	48	57	70	3	38	26.7	
9	Netherlands	NLD	16	166	9	151	171	25	11	12	-	173	4	27.0	
10	Denmark	DNK	70	181	3	125	178	4	12	52	9	180	16	27.4	
11	Sri Lanka	LKA	34	156	110	111	31	56	33	30	41	7	34	28.9	
12	Indonesia	IDN	74	118	153	153	5	76	62	59	79	12	14	29.3	
13	Israel	ISR	33	40	64	123	128	110	93	5	6	62	9	30.0	
14	Bangladesh	BGD	5	80	166	134	84	1	26	45	81	36	101	31.2	
15	Malta	MLT	4	154	21	36	-	214	127	69	2	138	3	34.0	
16	China	CHN	64	149	129	166	194	111	3	29	33	20	47	34.5	
17	New Zealand	NZL	177	128	6	113	98	89	73	13	91	1	93	35.4	
18	Iceland	ISL	207	144	2	44	128	195	13	2	106	-	-	36.9	
19	Honduras	HND	124	66	135	76	1	39	125	82	72	44	75	37.0	
20	DPR Korea	PRK	47	138	193	-	4	115	52	84	-	43	30	37.1	
