# Supplementary material for: Evaluating the Relative Environmental Impact of Countries
Source: PLoS One. 2010 May 3;5(5):e10440. doi: 10.1371/journal.pone.0010440 (PMC2862718; doi:10.1371/journal.pone.0010440)
Supplement: Table S3 — Twenty top-ranked countries by proportional composite environmental (pENV) rank (higher ranks = lower negative impact) when only two environmental variables were allowed to be missing (cf. three missing for rankings in main text and four missing in Table S4). Shown are country names and codes, population density (PD) rank, population growth rate (PGR) rank, governance quality (GOV) rank, Gross National Income (GNI) rank, natural forest loss (NFL) rank, natural habitat conversion (HBC) rank, marine captures (MC) rank, fertilizer use (FER) rank, water pollution (WTP) rank, proportion of threatened species (PTHR) rank, and carbon emissions (CO2) rank. Constituent variables used to create the pENV are in boldface. See text for details. Missing values denoted by ‘-’. (0.17 MB RTF) [file pone.0010440.s005.rtf]

Rank	Country	Code	PD	PGR	GOV	GNI	NFL	HBC	MC	FER	WTP	PTHR	CO2	pENV	
179	Cen Afr Rep	CAF	199	67	188	29	76	172	176.5	174	-	175	131	144.8	
178	Swaziland	SWZ	116	96	142	31	201	192	176.5	113	67	167	148	143.9	
177	Niger	NER	191	10	143	46	80	178	176.5	173	109	128	145	136.4	
176	Samoa	WSM	117	150	65	14	196	214	95	96	-	-	116	134.7	
175	Djibouti	DJI	153	53	151	19	128	184	152	-	-	98	109	130.8	
174	Tajikistan	TJK	137	119	182	38	161	124	176.5	111	-	93.5	-	129.6	
173	Bhutan	BTN	183	143	81	-	198	85	176.5	169	-	53	142	124.8	
172	Chad	TCD	197	12	181	41	70	112	176.5	148	-	125	144	124.3	
171	Mali	MLI	193	29	103	50	65	114	176.5	137	-	148	137	124.0	
170	Kazakhstan	KAZ	200	207	146	114	157	107	176.5	152	-	57	-	120.8	
169	Gabon	GAB	201	63	125	39	81	161	86	163	110	144	124	120.0	
168	Turkmenistan	TKM	192	91	189	70	128	182	176.5	90	-	66	-	119.6	
167	Lesotho	LSO	114	116	102	34	128	126	176.5	120	46	157	138	119.1	
166	Suriname	SUR	208	152	94	22	128	181	66	73	-	183	136	118.6	
165	Eritrea	ERI	148	52	168	27	77	117	148	133	-	132	-	118.5	
164	Canada	CAN	204	141	10	155	128	168	135	95	97	106	114	118.2	
163	Afghanistan	AFG	149	3	196	56	78	116	176.5	168	112	75	130	116.6	
162	Guin-Bissau	GNB	139	20	178	16	42	137	147	155	-	155	119	115.9	
161	Mozambique	MOZ	161	27	116	68	69	136	133	151	104	107	133	115.8	
160	Libya	LBY	203	79	169	-	128	190	84	103	-	84	126	114.3	
