# Supplementary material for: Evaluating the Relative Environmental Impact of Countries
Source: PLoS One. 2010 May 3;5(5):e10440. doi: 10.1371/journal.pone.0010440 (PMC2862718; doi:10.1371/journal.pone.0010440)
Supplement: Table S4 — Twenty top-ranked countries by proportional composite environmental (pENV) rank (higher ranks = lower negative impact) when four environmental variables were allowed to be missing (cf. three missing for rankings in main text and two missing in Table S3). Shown are country names and codes, population density (PD) rank, population growth rate (PGR) rank, governance quality (GOV) rank, Gross National Income (GNI) rank, natural forest loss (NFL) rank, natural habitat conversion (HBC) rank, marine captures (MC) rank, fertilizer use (FER) rank, water pollution (WTP) rank, proportion of threatened species (PTHR) rank, and carbon emissions (CO2) rank. Constituent variables used to create the pENV are in boldface. See text for details. Missing values denoted by ‘-’. (0.17 MB RTF) [file pone.0010440.s006.rtf]

Rank	Country	Code	PD	PGR	GOV	GNI	NFL	HBC	MC	FER	WTP	PTHR	CO2	pENV	
179	Liechtenstein	LIE	45	126	18	-	205	180	176.5	-	-	187	-	186.8	
178	Andorra	AND	59	72	19	-	128	189	176.5	-	-	178	-	166.0	
177	Cape Verde	CPV	69	54	76	20	128	214	113	157	-	-	-	148.5	
176	Cen Afr Rep	CAF	199	67	188	29	76	172	176.5	174	-	175	131	144.8	
175	Swaziland	SWZ	116	96	142	31	201	192	176.5	113	67	167	148	143.9	
174	Antig & Barb	ATG	50	85	52	9	128	148	119	-	-	176	-	141.1	
173	Martinique	MTQ	18	162	42	-	128	214	94	-	-	145.5	-	139.1	
172	Niger	NER	191	10	143	46	80	178	176.5	173	109	128	145	136.4	
171	Grenada	GRD	30	164	66	6	128	214	115	-	-	109	-	136.1	
170	Fr Guiana	GUF	211	13	59	-	86	186	116	-	-	181	-	135.4	
169	Samoa	WSM	117	150	65	14	196	214	95	96	-	-	116	134.7	
168	Kiribati	KIR	68	104	82	7	128	214	89	-	-	-	-	134.6	
167	Tonga	TON	66	185	109	8	128	214	132	-	-	-	88	133.6	
166	Bermuda	BMU	3	176	31	-	128	214	146	75	-	-	-	131.6	
165	Djibouti	DJI	153	53	151	19	128	184	152	-	-	98	109	130.8	
164	Tajikistan	TJK	137	119	182	38	161	124	176.5	111	-	93.5	-	129.6	
163	Bhutan	BTN	183	143	81	-	198	85	176.5	169	-	53	142	124.8	
162	Chad	TCD	197	12	181	41	70	112	176.5	148	-	125	144	124.3	
161	Vanuatu	VUT	172	48	88	4	128	165	81	-	-	-	139	124.2	
160	Mali	MLI	193	29	103	50	65	114	176.5	137	-	148	137	124.0	
