# Supplementary material for: Evaluating the Relative Environmental Impact of Countries
Source: PLoS One. 2010 May 3;5(5):e10440. doi: 10.1371/journal.pone.0010440 (PMC2862718; doi:10.1371/journal.pone.0010440)
Supplement: Table S5 — List of 49 missing countries from the proportional environmental impact ranking; minimum criterion for inclusion was ≤3 missing environmental variable values. (0.09 MB RTF) [file pone.0010440.s007.rtf]

American Samoa (ASM), Andorra (AND), Anguilla (AIA), Aruba (ABW), Bermuda (BMU), British Virgin Islands (VGB), Brunei Darussalam (BRN), Cayman Islands (CYM), Cook Islands (COK), Falkland Islands (FLK), French Guiana (GUF), French Polynesia (PYF), Gibraltar (GIB), Greenland (GRL), Guadeloupe (GLP), Guam (GUM), Guernsey (GGY), Isle of Man (IMN), Jersey (JEY), Kiribati (KIR), Liechtenstein (LIE), Maldives (MDV), Marshall Islands (MHL), Martinique (MTQ), Mayotte (MYT), Micronesia (FSM), Monaco (MCO), Montserrat (MSR), Nauru (NRU), Netherlands Antilles (ANT), New Caledonia (NCL), Niue (NIU), Norfolk Island (NFK), Northern Mariana Islands (MNP), Palau (PLW), Palestine (PSE), Pitcairn (PCN), Reunion (REU), Saint Helena (SHN), Saint Pierre and Miquelon (SPM), San Marino (SMR), Serbia and Montenegro (SCG), Svalbard and Jan Mayen (SJM), Taiwan (TWN), Timor-Leste (TLS), Tokelau (TKL), Turks and Caicos Islands (TCA), Tuvalu (TUV), Wallis and Futuna Islands (WLF)
