# Supplementary material for: Evaluating the Relative Environmental Impact of Countries
Source: PLoS One. 2010 May 3;5(5):e10440. doi: 10.1371/journal.pone.0010440 (PMC2862718; doi:10.1371/journal.pone.0010440)
Supplement: Table S6 — Full list of 179 countries ranked by proportional composite environmental (pENV) rank (lower ranks = higher negative impact). Shown are country names and codes, population density (PD) rank, population growth rate (PGR) rank, governance quality (GOV) rank, Gross National Income (GNI) rank, natural forest loss (NFL) rank, natural habitat conversion (HBC) rank, marine captures (MC) rank, fertilizer use (FER) rank, water pollution (WTP) rank, proportion of threatened species (PTHR) rank, and carbon emissions (CO2) rank. Constituent variables used to create the pENV are shaded. See text for details. Missing values denoted by ‘-’. (0.83 MB RTF) [file pone.0010440.s008.rtf]

Rank	Country	Code	PD	PGR	GOV	GNI	NFL	HBC	MC	FER	WTP	PTHR	CO2	pENV	
1	Singapore	SGP	1	51	13	115	128	5	91	1	4	63	1	10.6	
2	Rep Korea	KOR	14	158	56	154	23	61	20	17	21	29	5	20.4	
3	Qatar	QAT	108	8	67	-	-	198	112	20	3	-	7	24.8	
4	Kuwait	KWT	61	110	74	109	128	197	114	11	1	-	8	25.1	
5	Japan	JPN	23	188	30	165	87	87	18	21	29	13	6	25.2	
6	Thailand	THA	71	145	90	148	43	8	7	67	-	37	46	25.5	
7	Bahrain	BHR	6	41	73	52	-	193	59	4	-	123	2	25.7	
8	Malaysia	MYS	102	60	71	131	47	75	22	8	77	15	11	25.9	
9	Philippines	PHL	36	70	122	144	22	20	48	57	70	3	38	26.7	
10	Netherlands	NLD	16	166	9	151	171	25	11	12	-	173	4	27.0	
11	Denmark	DNK	70	181	3	125	178	4	12	52	9	180	16	27.4	
12	Sri Lanka	LKA	34	156	110	111	31	56	33	30	41	7	34	28.9	
13	Indonesia	IDN	74	118	153	153	5	76	62	59	79	12	14	29.3	
14	Israel	ISR	33	40	64	123	128	110	93	5	6	62	9	30.0	
15	Bangladesh	BGD	5	80	166	134	84	1	26	45	81	36	101	31.2	
16	Malta	MLT	4	154	21	36	-	214	127	69	2	138	3	34.0	
17	China	CHN	64	149	129	166	194	111	3	29	33	20	47	34.5	
18	New Zealand	NZL	177	128	6	113	98	89	73	13	91	1	93	35.4	
19	Iceland	ISL	207	144	2	44	128	195	13	2	106	-	-	36.9	
20	Honduras	HND	124	66	135	76	1	39	125	82	72	44	75	37.0	
21	DPR Korea	PRK	47	138	193	-	4	115	52	84	-	43	30	37.1	
22	Ecuador	ECU	136	106	148	99	11	78	30	55	98	22	56	40.2	
23	Poland	POL	72	195	63	146	184	27	15	63	14	133	21	40.7	
24	Solomon Is	SLB	174	32	154	13	2	183	134	-	-	17	134	40.7	
25	Peru	PER	168	111	120	119	61	159	1	87	107	27	80	41.0	
26	Cambodia	KHM	104	50	155	82	7	43	64	156	-	30	54	41.2	
27	Germany	DEU	44	186	16	163	183	29	46	39	16	141	10	41.6	
28	Guatemala	GTM	75	55	139	101	17	47	74	60	89	25	31	42.4	
29	Liberia	LBR	157	16	194	-	19	17	111	-	-	80	48	42.4	
30	Costa Rica	CRI	97	62	57	90	46	40	103	7	75	49	55	42.9	
31	Viet Nam	VNM	39	103	134	127	203	13	23	26	-	28	147	43.2	
32	India	IND	21	90	106	164	179	32	17	70	51	14	65	43.9	
33	Jamaica	JAM	42	151	98	51	83	48	100	76	39	16	18	44.4	
34	Mexico	MEX	131	115	93	156	51	121	51	88	60	4	70	46.4	
35	Myanmar	MMR	111	132	197	-	12	35	43	132	111	55	32	46.4	
36	Spain	ESP	96	157	29	157	206	52	21	48	28	50	35	47.3	
37	Ghana	GHA	91	47	99	97	16	79	4	146	78	112	96	48.4	
38	El Salvador	SLV	27	95	104	87	45	9	79	65	57	135	42	48.9	
39	South Africa	ZAF	147	93	72	147	92	105	16	97	20	47	57	50.2	
40	Lithuania	LTU	132	206	45	95	189	23	6	80	42	154	63	50.6	
41	Morocco	MAR	113	121	107	118	96	104	5	101	31	56	110	51.5	
42	UK	GBR	41	182	14	162	180	84	65	25	22	159	12	52.0	
43	France	FRA	79	172	24	161	188	44	35	38	30	115	28	52.2	
44	Cyprus	CYP	95	122	36	61	185	59	63	44	12	119	26	52.7	
45	Lebanon	LBN	17	82	127	71	176	54	104	31	27	83	17	53.0	
46	Ukraine	UKR	103	208	137	141	169	14	42	124	25	72	-	53.0	
47	Czech Republic	CZE	67	198	43	130	165	33	176.5	64	10	163	13	53.8	
48	Italy	ITA	48	189	48	160	204	60	75	42	32	82	15	55.0	
49	Mauritius	MUS	10	137	53	53	82	214	82	27	13	-	-	55.0	
50	Dominica	DMA	94	199	50.5	2	34	187	121	6	-	117	-	55.8	
51	Haiti	HTI	24	94	192	67	73	49	138	126	-	5	98	55.9	
52	Madagascar	MDG	155	21	101	63	63	101	108	158	86	2	104	56.9	
53	Pakistan	PAK	46	65	170	140	71	80	19	56	66	58	85	57.0	
54	Sierra Leone	SLE	101	75	174	25	41	10	61	162	-	114	78	57.5	
55	USA	USA	156	139	20	167	91	103	68	68	55	24	37	57.7	
56	Belgium	BEL	22	184	17	142	173	18	176.5	22	17	179	-	57.7	
57	Cuba	CUB	86	178	161	-	202	28	139	105	-	6	76	57.9	
58	Portugal	PRT	77	180	26	132	128	90	38	49	38	76	36	58.1	
59	Senegal	SEN	125	36	105	69	40	38	10	123	83	121	121	58.2	
60	Dominican Rep	DOM	49	99	108	103	128	70	118	66	-	10	62	59.2	
61	Nigeria	NGA	60	34	184	117	25	55	31	144	76	95	58	59.3	
62	Armenia	ARM	87	214	118	49	55	12	176.5	121	44	70	-	59.3	
63	Hungary	HUN	83	201	39	120	174	6	176.5	74	48	127	33	59.8	
64	Nicaragua	NIC	143	88	126	66	13	51	110	119	-	137	39	59.9	
65	Jordan	JOR	121	9	89	79	128	191	129	18	8	73	86	60.2	
66	Panama	PAN	141	84	85	73	58	62	67	94	100	59	22	60.4	
67	Venezuela	VEN	159	81	175	124	37	119	44	61	102	48	51	60.5	
68	Brazil	BRA	166	114	95	159	35	83	99	62	101	23	74	60.8	
69	Bulgaria	BGR	112	211	78	107	195	31	85	81	19	96	44	61.5	
70	Colombia	COL	146	102	138	139	74	93	87	33	103	19	90	61.9	
71	Seychelles	SYC	51	133	84	17	128	214	36	116	-	8	-	62.0	
72	Papua New Guinea	PNG	184	42	150	58	38	102	123	72	-	33	52	62.4	
73	Nepal	NPL	52	59	164	92	20	77	176.5	114	95	54	24	62.7	
74	Turkey	TUR	92	105	100	150	89	22	176.5	83	52	42	66	63.4	
75	Slovenia	SVN	89	187	35	94	199	100	69	19	45	142	25	63.5	
76	Togo	TGO	81	18	172	40	32	133	8	143	-	160.5	84	63.5	
77	Laos	LAO	164	69	177	42	29	24	176.5	138	-	39	103	63.9	
78	Ireland	IRL	127	136	15	116	97	162	60	14	59	150	40	64.6	
79	US Virgin Is	VIR	26	170	38	-	30	88	142	24	-	126	-	64.7	
80	Saint Lucia	LCA	37	140	49	11	128	214	109	3	-	129	-	64.9	
81	Egypt	EGY	107	87	131	137	128	167	83	16	26	68	100	65.2	
82	Benin	BEN	106	14	113	37	15	122	41	129	-	158	53	65.8	
83	Chile	CHL	167	117	23	122	95	149	47	36	99	21	108	65.9	
84	Trin & Tob	TTO	38	169	77	55	59	98	96	28	35	151	-	65.9	
85	Puerto Rico	PRI	15	155	37	-	170	37	143	-	36	40	-	66.4	
86	St Vincent Gren	VCT	32	167	55	5	128	214	34	9	-	160.5	-	67.0	
87	Zimbabwe	ZWE	154	112	195	91	9	81	176.5	102	43	130	91	67.9	
88	UAE	ARE	134	1	61	112	128	196	70	10	-	85	-	68.4	
89	Argentina	ARG	181	134	121	149	68	118	25	109	82	38	111	69.2	
90	Barbados	BRB	8	171	28	105	128	214	77	50	7	162	-	70.2	
91	Cameroon	CMR	150	45	160	86	24	129	71	140	105	34	82	70.9	
92	Tunisia	TUN	122	120	87	108	181	142	54	106	11	64	94	71.7	
93	Greece	GRC	98	165	46	133	197	72	122	46	63	67	29	71.7	
94	Bahamas	BHS	165	107	27	30	128	169	150	91	5	102	69	72.4	
95	Latvia	LVA	152	212	50.5	80	191	57	14	93	53	172	81	72.4	
96	Oman	OMN	196	78	68	89	128	188	56	34	24	86	113	72.7	
97	Eq Guinea	GNQ	173	57	185	10	18	128	128	-	-	100	72	73.4	
98	Romania	ROU	93	205	91	128	99	30	136	107	71	93.5	41	73.7	
99	Tanzania	TZA	144	31	124	77	27	96	98	149	73	35	129	74.3	
100	Cote dIvoire	CIV	129	44	187	84	128	53	40	117	94	71	61	74.7	
101	Algeria	DZA	182	98	152	129	162	185	37	128	15	52	123	75.2	
102	Sao Tome & Princ	STP	57	89	112	1	128	214	97	-	-	9	107	76.1	
103	Croatia	HRV	99	194	75	100	166	50	131	53	68	79	49	76.4	
104	Uruguay	URY	171	175	60	88	163	74	27	77	92	45	149	76.6	
105	Moldova	MDA	76	210	140	47	167	15	176.5	125	37	99	-	76.6	
106	Belarus	BLR	135	204	179	-	190	41	-	51	-	147	50	78.2	
107	Namibia	NAM	210	56	80	57	64	177	9	165	-	101	143	79.0	
108	Macedonia	MKD	100	177	123	60	128	45	176.5	100	23	108	-	79.5	
109	Switzerland	CHE	53	168	5	138	187	147	176.5	37	34	174	19	79.6	
110	Fiji	FJI	138	148	97	28	128	135	117	92	87	11	115	79.7	
111	Iraq	IRQ	120	30	199	-	159	173	29	85	-	46	83	79.8	
112	Bos & Herz	BIH	105	209	128	-	48	36	153	108	88	124	67	79.9	
113	Belize	BLZ	188	39	83	18	128	123	101	54	-	153	20	80.0	
114	Yemen	YEM	145	7	173	62	128	160	49	141	18	65	128	80.1	
115	Slovakia	SVK	80	191	47	110	164	58	176.5	79	49	143	23	80.2	
116	Syria	SYR	85	38	159	106	128	139	102	86	50	41	68	80.4	
117	Iran	IRN	142	123	163	145	128	163	57	89	47	60	77	81.1	
118	Estonia	EST	158	213	33	65	193	63	72	41	-	177	45	81.2	
119	Burundi	BDI	35	71	186	32	21	73	176.5	161	-	120	64	83.3	
120	Australia	AUS	209	127	11	152	79	145	141	99	84	18	118	83.6	
121	Russia	RUS	194	202	141	158	88	146	76	127	74	31	105	84.1	
122	Austria	AUT	88	174	12	135	207	108	176.5	43	54	122	27	84.3	
123	Guinea	GIN	151	35	171	72	53	92	50	160	-	91	117	86.4	
124	Zambia	ZMB	176	61	136	48	14	140	176.5	139	-	164	59	88.0	
125	Luxembourg	LUX	55	130	4	74	168	64	176.5	15	-	187	-	88.2	
126	Comoros	COM	19	28	162	15	54	95	78	153	-	-	-	88.5	
127	Malawi	MWI	78	64	133	35	36	91	176.5	118	62	140	73	88.7	
128	Mauritania	MRT	206	25	115	33	93	175	24	145	-	103	-	89.8	
129	Gambia	GMB	63	11	119	21	192	21	45	147	-	149	146	91.3	
130	St Kitts & Nevis	KNA	65	131	54	3	128	94	-	35	-	168	-	91.7	
131	Paraguay	PRY	179	68	156	83	26	109	176.5	110	-	105	112	93.0	
132	Rwanda	RWA	20	108	157	43	85	46	176.5	171	-	92	60	93.1	
133	Uganda	UGA	73	15	144	85	33	67	176.5	167	-	116	87	93.3	
134	Norway	NOR	180	163	8	126	175	154	53	40	93	134	102	95.5	
135	Ethiopia	ETH	110	23	167	104	56	69	176.5	130	90	69	135	95.9	
136	Burkina Faso	BFA	133	19	117	54	62	26	176.5	142	85	156	140	96.0	
137	Kenya	KEN	123	26	149	93	90	132	130	112	40	97	125	97.5	
138	Georgia	GEO	118	215	145	59	94	97	126	104	-	77	-	98.3	
139	Angola	AGO	185	24	183	75	60	166	32	164	-	139	127	98.7	
140	DRC	COD	162	22	198	96	52	158	80	172	-	88	97	99.4	
141	Congo	COG	190	37	180	24	75	164	28	136	-	169	122	99.4	
142	Albania	ALB	82	203	130	-	177	42	140	98	96	131	92	102.4	
143	Azerbaijan	AZE	90	146	165	81	128	86	176.5	135	58	78	-	102.9	
144	Sudan	SDN	178	58	191	102	49	99	144	154	64	152	132	104.7	
145	Mongolia	MNG	212	142	92	26	72	127	176.5	159	80	51	141	105.7	
146	Uzbekistan	UZB	126	100	190	98	172	155	176.5	47	-	61	-	106.2	
147	Saudi Arabia	SAU	189	46	111	143	128	194	120	78	-	74	95	108.5	
148	Bolivia	BOL	195	73	132	78	44	150	176.5	150	108	89	106	108.6	
149	Kyrgyzstan	KGZ	160	135	158	45	160	106	176.5	122	56	87	-	110.1	
150	Sweden	SWE	169	179	7	136	158	143	106	71	65	182	99	110.3	
151	Finland	FIN	175	183	1	121	182	151	137	58	61	171	89	110.6	
152	Botswana	BWA	205	83	44	64	50	141	176.5	131	69	166	120	112.2	
153	Guyana	GUY	202	193	114	23	128	179	58	115	-	170	79	112.7	
154	Libya	LBY	203	79	169	-	128	190	84	103	-	84	126	114.3	
155	Mozambique	MOZ	161	27	116	68	69	136	133	151	104	107	133	115.8	
156	Guin-Bissau	GNB	139	20	178	16	42	137	147	155	-	155	119	115.9	
157	Somalia	SOM	186	124	200	-	57	156	124	170	-	113	-	116.2	
158	Afghanistan	AFG	149	3	196	56	78	116	176.5	168	112	75	130	116.6	
159	Canada	CAN	204	141	10	155	128	168	135	95	97	106	114	118.2	
160	Eritrea	ERI	148	52	168	27	77	117	148	133	-	132	-	118.5	
161	Suriname	SUR	208	152	94	22	128	181	66	73	-	183	136	118.6	
162	Lesotho	LSO	114	116	102	34	128	126	176.5	120	46	157	138	119.1	
163	Turkmenistan	TKM	192	91	189	70	128	182	176.5	90	-	66	-	119.6	
164	Gabon	GAB	201	63	125	39	81	161	86	163	110	144	124	120.0	
165	Kazakhstan	KAZ	200	207	146	114	157	107	176.5	152	-	57	-	120.8	
166	Mali	MLI	193	29	103	50	65	114	176.5	137	-	148	137	124.0	
167	Vanuatu	VUT	172	48	88	4	128	165	81	-	-	-	139	124.2	
168	Chad	TCD	197	12	181	41	70	112	176.5	148	-	125	144	124.3	
169	Bhutan	BTN	183	143	81	-	198	85	176.5	169	-	53	142	124.8	
170	Tajikistan	TJK	137	119	182	38	161	124	176.5	111	-	93.5	-	129.6	
171	Djibouti	DJI	153	53	151	19	128	184	152	-	-	98	109	130.8	
172	Tonga	TON	66	185	109	8	128	214	132	-	-	-	88	133.6	
173	Samoa	WSM	117	150	65	14	196	214	95	96	-	-	116	134.7	
174	Grenada	GRD	30	164	66	6	128	214	115	-	-	109	-	136.1	
175	Niger	NER	191	10	143	46	80	178	176.5	173	109	128	145	136.4	
176	Antig & Barb	ATG	50	85	52	9	128	148	119	-	-	176	-	141.1	
177	Swaziland	SWZ	116	96	142	31	201	192	176.5	113	67	167	148	143.9	
178	Cen Afr Rep	CAF	199	67	188	29	76	172	176.5	174	-	175	131	144.8	
179	Cape Verde	CPV	69	54	76	20	128	214	113	157	-	-	-	148.5	
