# Supplementary material for: Evaluating the Relative Environmental Impact of Countries
Source: PLoS One. 2010 May 3;5(5):e10440. doi: 10.1371/journal.pone.0010440 (PMC2862718; doi:10.1371/journal.pone.0010440)
Supplement: Table S8 — Full list of 171 countries ranked by absolute composite environmental (aENV) rank (lower ranks = higher negative impact). Shown are country names and codes, population density (PD) rank, population growth rate (PGR) rank, governance quality (GOV) rank, Gross National Income (GNI) rank, natural forest loss (NFL) rank, natural habitat conversion (HBC) rank, marine captures (MC) rank, fertilizer use (FER) rank, water pollution (WTP) rank, proportion of threatened species (PTHR) rank, and carbon emissions (CO2) rank. Constituent variables used to create the aENV are shaded. See text for details. Missing values denoted by ‘-’. (0.82 MB RTF) [file pone.0010440.s010.rtf]

Rank	Country	Code	PD	PGR	GOV	GNI	NFL	HBC	MC	FER	WTP	PTHR	CO2	aENV	
1	Brazil	BRA	166	114	95	159	1	3	30	3	8	4	4	4.5	
2	USA	USA	156	139	20	167	21	211.5	3	1	2	9	1	5.9	
3	China	CHN	64	149	129	166	216	36	1	-	1	6	2	6.7	
4	Indonesia	IDN	74	118	153	153	2	183	6	6	7	3	3	7.0	
5	Japan	JPN	23	188	30	165	73	5	4	17	5	23.5	6	10.8	
6	Mexico	MEX	131	115	93	156	9	211.5	17	13	17	1	12	13.6	
7	India	IND	21	90	106	164	214	137	8	2	3	8	8	13.7	
8	Russia	RUS	194	202	141	158	12	125	7	18	4	26	5	13.9	
9	Australia	AUS	209	127	11	152	10	7	47	9	31	11.5	18	15.2	
10	Peru	PER	168	111	120	119	27	30	2	46	49	7	27	18.3	
11	Argentina	ARG	181	134	121	149	19	11	21	23	22	16	31	19.6	
12	Canada	CAN	204	141	10	155	133.5	6	19	7	16	71	10	19.8	
13	Malaysia	MYS	102	60	71	131	39	170	16	22	24	10	9	24.3	
14	Myanmar	MMR	111	132	197	-	4	18	22	113	102	25	14	25.2	
15	Ukraine	UKR	103	208	137	141	201	1	39	36	11	90	-	25.6	
16	Thailand	THA	71	145	90	148	28	211.5	9	11	-	20	29	26.4	
17	Philippines	PHL	36	70	122	144	22	168	12	27	21	11.5	33	26.6	
18	France	FRA	79	172	24	161	210	-	26	4	9	116.5	16	26.7	
19	South Africa	ZAF	147	93	72	147	63	43	25	28	19	31	17	29.4	
20	Colombia	COL	146	102	138	139	43	162	64	30	30	2	32	30.7	
21	Ecuador	ECU	136	106	148	99	16	86	32	54	60	5	44	31.6	
22	Venezuela	VEN	159	81	175	124	13	173	33	40	36	14	22	31.8	
23	Rep Korea	KOR	14	158	56	154	49	192	13	29	15	50.5	15	32.7	
24	Tanzania	TZA	144	31	124	77	8	9	79	80	63	15	99	33.0	
25	Pakistan	PAK	46	65	170	140	50	98	34	5	34	45.5	35	33.3	
26	Nigeria	NGA	60	34	184	117	7	70	40	57	39	43	25	33.4	
27	Chile	CHL	167	117	23	122	76	72	5	33	42	32.5	52	35.0	
28	Papua New Guin	PNG	184	42	150	58	23	23	56	87	-	22	34	35.3	
29	Germany	DEU	44	186	16	163	204	166	45	8	6	140.5	7	35.6	
30	DRC	COD	162	22	198	96	5	121	132	-	-	34	21	35.6	
31	Turkey	TUR	92	105	100	150	64	129	31	12	23	50.5	30	37.6	
32	UK	GBR	41	182	14	162	198	111	24	16	10	158	11	39.4	
33	Bangladesh	BGD	5	80	166	134	78	39	36	19	18	40.5	98	39.4	
34	Romania	ROU	93	205	91	128	99.5	2	149	43	41	106	39	41.6	
35	Cameroon	CMR	150	45	160	86	15	42	73	82	86	18	46	42.9	
36	Bolivia	BOL	195	73	132	78	14	63	-	103	84	23.5	40	43.9	
37	Spain	ESP	96	157	29	157	215	92	20	-	14	60	23	44.4	
38	Italy	ITA	48	189	48	160	212	136	41	21	12	90	13	44.6	
39	Iran	IRN	142	123	163	145	133.5	120	43	25	26	52	19	46.1	
40	Saudi Arabia	SAU	189	46	111	143	133.5	12	77	44	-	90	24	47.7	
41	Hungary	HUN	83	201	39	120	188	8	-	34	29	140.5	58	47.9	
42	Egypt	EGY	107	87	131	137	133.5	109	58	15	20	76	37	49.3	
43	Viet Nam	VNM	39	103	134	127	213	211.5	14	10	-	17	148	50.1	
44	Poland	POL	72	195	63	146	203	140	48	20	13	140.5	20	51.8	
45	Sri Lanka	LKA	34	156	110	111	57	139	44	50	40	21	73	52.3	
46	DPR Korea	PRK	47	138	193	-	26	94	50	-	-	68.5	47	52.4	
47	New Zealand	NZL	177	128	6	113	95	150	28	26	51	28	76	52.7	
48	Czech Republic	CZE	67	198	43	130	179.5	20	-	41	25	153	38	52.7	
49	Chad	TCD	197	12	181	41	36	17	-	-	-	106	129	53.8	
50	Sudan	SDN	178	58	191	102	3	159	130	76	57	76	78	55.4	
51	Madagascar	MDG	155	21	101	63	45	181	66	116	45	13	56	57.4	
52	Honduras	HND	124	66	135	76	17	87	108	85	61	35	87	59.1	
53	Morocco	MAR	113	121	107	118	83	184	23	37	38	68.5	80	59.7	
54	Laos	LAO	164	69	177	42	33	135	-	-	-	32.5	88	59.7	
55	Ethiopia	ETH	110	23	167	104	24	84	-	74	72	39	112	60.1	
56	Paraguay	PRY	179	68	156	83	18	157	-	55	-	60	84	60.1	
57	Zimbabwe	ZWE	154	112	195	91	11	211.5	-	67	65	82.5	57	60.2	
58	Guatemala	GTM	75	55	139	101	44	154	104	60	73	19	49	60.2	
59	Portugal	PRT	77	180	26	132	133.5	56	46	48	27	102.5	64	60.4	
60	Uganda	UGA	73	15	144	85	35	60	-	118	-	48	72	61.2	
61	Nepal	NPL	52	59	164	92	37	211.5	-	89	67	36	36	62.7	
62	Israel	ISR	33	40	64	123	133.5	33	139	31	47	71	61	62.8	
63	Costa Rica	CRI	97	62	57	90	66	65	90	61	62	27	101	63.1	
64	Netherlands	NLD	16	166	9	151	182	85	29	32	-	170	26	63.2	
65	Angola	AGO	185	24	183	75	29	80	51	117	-	65.5	83	65.0	
66	Norway	NOR	180	163	8	126	199	77	10	62	48	148.5	75	65.3	
67	Belarus	BLR	135	204	179	-	208	32	-	24	-	153	50	65.7	
68	Cambodia	KHM	104	50	155	82	20	211.5	81	109	-	37.5	60	66.2	
69	Denmark	DNK	70	181	3	125	185	113	15	52	37	177	55	66.7	
70	Ireland	IRL	127	136	15	116	99.5	54	42	35	59	163	79	66.9	
71	Trin & Tob	TTO	38	169	77	55	85.5	10	112	84	92	132.5	-	67.9	
72	Ghana	GHA	91	47	99	97	25	192	37	94	82	60	82	68.0	
73	Algeria	DZA	182	98	152	129	206	57	61	69	43	73.5	45	68.4	
74	Kenya	KEN	123	26	149	93	69	79	122	56	50	40.5	95	68.6	
75	Syria	SYR	85	38	159	106	133.5	26	146	42	80	71	67	69.8	
76	Cote d'Ivoire	CIV	129	44	187	84	133.5	68	76	73	89	44	42	70.1	
77	Malawi	MWI	78	64	133	35	53	45	-	78	88	90	85	70.7	
78	Panama	PAN	141	84	85	73	72	211.5	49	97	85	29.5	51	71.2	
79	Estonia	EST	158	213	33	65	192	13	68	51	-	177	92	72.1	
80	Serb & Mont	SCG	84	190	-	-	193	46	164	14	-	106	66	72.3	
81	Iceland	ISL	207	144	2	44	133.5	141	11	101	96	-	-	72.5	
82	Bulgaria	BGR	112	211	78	107	205	55	118	38	33	106	65	73.4	
83	Haiti	HTI	24	94	192	67	81	47	123	-	-	37.5	128	74.2	
84	Afghanistan	AFG	149	3	196	56	55	38	-	-	115	82.5	114	74.3	
85	Namibia	NAM	210	56	80	57	40	156	27	128	-	63	127	74.6	
86	Botswana	BWA	205	83	44	64	30	78	-	-	100	132.5	89	77.3	
87	Eritrea	ERI	148	52	168	27	74	21	125	131	-	111.5	-	77.7	
88	Oman	OMN	196	78	68	89	133.5	22	57	102	99	111.5	93	78.0	
89	Liberia	LBR	157	16	194	-	42	131	120	-	-	60	74	78.2	
90	Nicaragua	NIC	143	88	126	66	32	211.5	101	77	-	85	54	78.9	
91	Kazakhstan	KAZ	200	207	146	114	187	61	-	64	-	55.5	-	79.8	
92	Greece	GRC	98	165	46	133	207	211.5	67	39	55	79	43	80.2	
93	Macedonia	MKD	100	177	123	60	133.5	28	-	112	69	124.5	-	81.5	
94	Sweden	SWE	169	179	7	136	178	211.5	38	49	35	180.5	59	82.6	
95	Mozambique	MOZ	161	27	116	68	48	104	91	95	90	60	113	82.6	
96	Solomon Is	SLB	174	32	154	13	52	122	83	-	-	53.5	138	82.8	
97	Austria	AUT	88	174	12	135	211	108	-	45	46	132.5	53	83.2	
98	Burkina Faso	BFA	133	19	117	54	56	34	-	92	106	132.5	135	83.2	
99	Senegal	SEN	125	36	105	69	46	211.5	35	83	93	90	122	83.7	
100	Slovakia	SVK	80	191	47	110	173.5	59	-	65	52	145	70	84.0	
101	Kuwait	KWT	61	110	74	109	133.5	37	126	105	87	-	63	84.3	
102	Uruguay	URY	171	175	60	88	186	91	60	63	71	55.5	149	86.9	
103	Guinea	GIN	151	35	171	72	51	167	70	126	-	53.5	111	87.4	
104	Finland	FIN	175	183	1	121	202	149	62	47	44	170	62	87.9	
105	Jordan	JOR	121	9	89	79	133.5	40	176	58	74	99	102	88.0	
106	Gabon	GAB	201	63	125	39	68	44	84	132	109	99	117	88.3	
107	Mongolia	MNG	212	142	92	26	34	161	-	125	94	65.5	119	89.1	
108	Yemen	YEM	145	7	173	62	133.5	66	52	122	83	90	109	89.4	
109	Sierra Leone	SLE	101	75	174	25	59	178	72	-	-	73.5	105	89.8	
110	Eq Guinea	GNQ	173	57	185	10	61	73	141	-	-	76	125	90.2	
111	Mauritania	MRT	206	25	115	33	70	187	54	-	-	95.5	-	90.6	
112	El Salvador	SLV	27	95	104	87	75	89	100	79	76	111.5	115	90.9	
113	Latvia	LVA	152	212	50.5	80	197	51	59	71	66	170	110	91.1	
114	Moldova	MDA	76	210	140	47	172	41	-	99	68	132.5	-	91.1	
115	Cuba	CUB	86	178	161	-	209	185	80	72	-	29.5	91	91.8	
116	Switzerland	CHE	53	168	5	138	189	143	-	70	28	170	68	92.2	
117	Benin	BEN	106	14	113	37	41	117	116	114	-	128	77	92.5	
118	Dominican Rep	DOM	49	99	108	103	133.5	101	109	-	-	45.5	104	93.0	
119	Guyana	GUY	202	193	114	23	133.5	74	78	110	-	111.5	71	93.6	
120	Croatia	HRV	99	194	75	100	177	123	96	59	54	102.5	94	93.9	
121	Rwanda	RWA	20	108	157	43	85.5	138	-	-	-	57	121	95.0	
122	Malta	MLT	4	154	21	36	-	16	158	133	104	163	131	95.2	
123	Mauritius	MUS	10	137	53	53	99.5	95	111	98	77	-	-	95.4	
124	Armenia	ARM	87	214	118	49	77	105	-	111	91	99	-	95.8	
125	Azerbaijan	AZE	90	146	165	81	133.5	96	-	96	70	95.5	-	96.2	
126	Kyrgyzstan	KGZ	160	135	158	45	176	52	-	104	79	111.5	-	96.5	
127	Tunisia	TUN	122	120	87	108	195	211.5	69	66	53	90	90	97.0	
128	Lithuania	LTU	132	206	45	95	194	151	63	53	56	158	97	97.5	
129	Belgium	BEL	22	184	17	142	179.5	99	88	-	32	177	-	97.6	
130	Mali	MLI	193	29	103	50	31	211.5	-	-	-	116.5	120	97.8	
131	Singapore	SGP	1	51	13	115	133.5	211.5	133	119	64	79	41	98.9	
132	Togo	TGO	81	18	172	40	58	103	102	107	-	124.5	116	99.0	
133	Jamaica	JAM	42	151	98	51	89	171	107	115	75	65.5	106	99.6	
134	Samoa	WSM	117	150	65	14	171	25	121	136	-	-	141	99.8	
135	Cen Afr Rep	CAF	199	67	188	29	54	115	-	-	-	136.5	118	100.0	
136	Iraq	IRQ	120	30	199	-	182	169	114	-	-	65.5	48	102.0	
137	Burundi	BDI	35	71	186	32	62	128	-	129	-	90	124	102.7	
138	Bosnia & Herz	BIH	105	209	128	-	67	88	181	86	97	140.5	107	104.3	
139	Libya	LBY	203	79	169	-	133.5	211.5	82	68	-	120.5	69	104.6	
140	Lebanon	LBN	17	82	127	71	170	71	138	90	81	120.5	103	106.0	
141	Cyprus	CYP	95	122	36	61	173.5	67	92	100	95	145	123	108.9	
142	Puerto Rico	PRI	15	155	37	-	169	102	142	-	78	82.5	-	109.5	
143	Belize	BLZ	188	39	83	18	133.5	145	89	106	-	120.5	86	111.2	
144	United Arab Em	ARE	134	1	61	112	133.5	180	65	88	-	124.5	-	111.3	
145	Congo	COG	190	37	180	24	60	211.5	99	-	-	128	108	111.7	
146	Niger	NER	191	10	143	46	47	211.5	-	121	111	120.5	133	113.5	
147	Dominica	DMA	94	199	50.5	2	90	75	161	-	-	153	-	113.6	
148	Lesotho	LSO	114	116	102	34	133.5	64	-	-	105	158	140	114.7	
149	Seychelles	SYC	51	133	84	17	133.5	177	75	-	-	99	-	115.1	
150	Georgia	GEO	118	215	145	59	87.5	174	150	93	-	106	-	117.6	
151	Qatar	QAT	108	8	67	-	-	211.5	119	108	103	-	81	117.8	
152	Guinea-Bissau	GNB	139	20	178	16	71	144	124	-	-	132.5	136	118.0	
153	Slovenia	SVN	89	187	35	94	190	175	156	75	58	145	100	118.5	
154	Fiji	FJI	138	148	97	28	133.5	155	85	120	98	116.5	137	118.6	
155	Sao Tome & Princ	STP	57	89	112	1	133.5	97	134	-	-	111.5	143	122.6	
156	Luxembourg	LUX	55	130	4	74	166.5	83	-	91	-	187	-	123.8	
157	Albania	ALB	82	203	130	-	182	114	152	81	101	140.5	126	124.2	
158	Suriname	SUR	208	152	94	22	133.5	134	95	123	-	153	134	127.4	
159	Bahamas	BHS	165	107	27	30	133.5	160	113	-	110	136.5	130	129.5	
160	Vanuatu	VUT	172	48	88	4	133.5	211.5	71	-	-	-	144	130.3	
161	Bahrain	BHR	6	41	73	52	-	194.5	110	124	-	158	96	132.1	
162	Tonga	TON	66	185	109	8	133.5	119	137	-	-	-	142	132.6	
163	St Kitts & Nevis	KNA	65	131	54	3	133.5	81	165	-	-	177	-	133.3	
164	Gambia	GMB	63	11	119	21	184	146	87	-	-	128	146	134.3	
165	St Vincent Gren	VCT	32	167	55	5	133.5	186	97	-	114	170	-	136.1	
166	Swaziland	SWZ	116	96	142	31	191	112	-	-	108	140.5	147	136.7	
167	Barbados	BRB	8	171	28	105	133.5	211.5	147	130	107	170	-	146.3	
168	Djibouti	DJI	153	53	151	19	133.5	190	169	-	-	124.5	132	147.8	
169	Grenada	GRD	30	164	66	6	133.5	188	153	-	112	170	-	148.9	
170	Saint Lucia	LCA	37	140	49	11	133.5	192	155	-	-	158	-	158.3	
171	Antig & Barb	ATG	50	85	52	9	133.5	182	151	-	-	180.5	-	160.4	
