# Supplementary material for: Evaluating the Relative Environmental Impact of Countries
Source: PLoS One. 2010 May 3;5(5):e10440. doi: 10.1371/journal.pone.0010440 (PMC2862718; doi:10.1371/journal.pone.0010440)
Supplement: Table S9 — Kendall's rank correlation (τ) matrix for socio-economic ranks: POP = human population size (2005), POPD = human population density (2005), PGR = human population growth rate (1990–2005), GNI = purchasing power parity-adjusted Gross National Income, GOV = governance quality. Lower-left quadrant values are Kendall's τ; upper-right quadrant values are Type I error probabilities for the coefficients. Boldface τ indicate sufficient evidence of a relationship. (0.09 MB RTF) [file pone.0010440.s011.rtf]

	POP	POPD	PGR	GNI	GOV	
POP	-	0.7159	0.5601	<0.0001	0.0054	
POPD	0.019	-	0.0125	0.1075	0.0086	
PGR	0.030	-0.130	-	0.0003	<0.0001	
GNI	0.622	0.084	-0.187	-	0.0020	
GOV	-0.145	0.137	-0.358	0.161	-	
