# Supplementary figures and images for: Evaluating the Relative Environmental Impact of Countries
Source: PLoS One. 2010 May 3;5(5):e10440. doi: 10.1371/journal.pone.0010440 (PMC2862718; doi:10.1371/journal.pone.0010440)

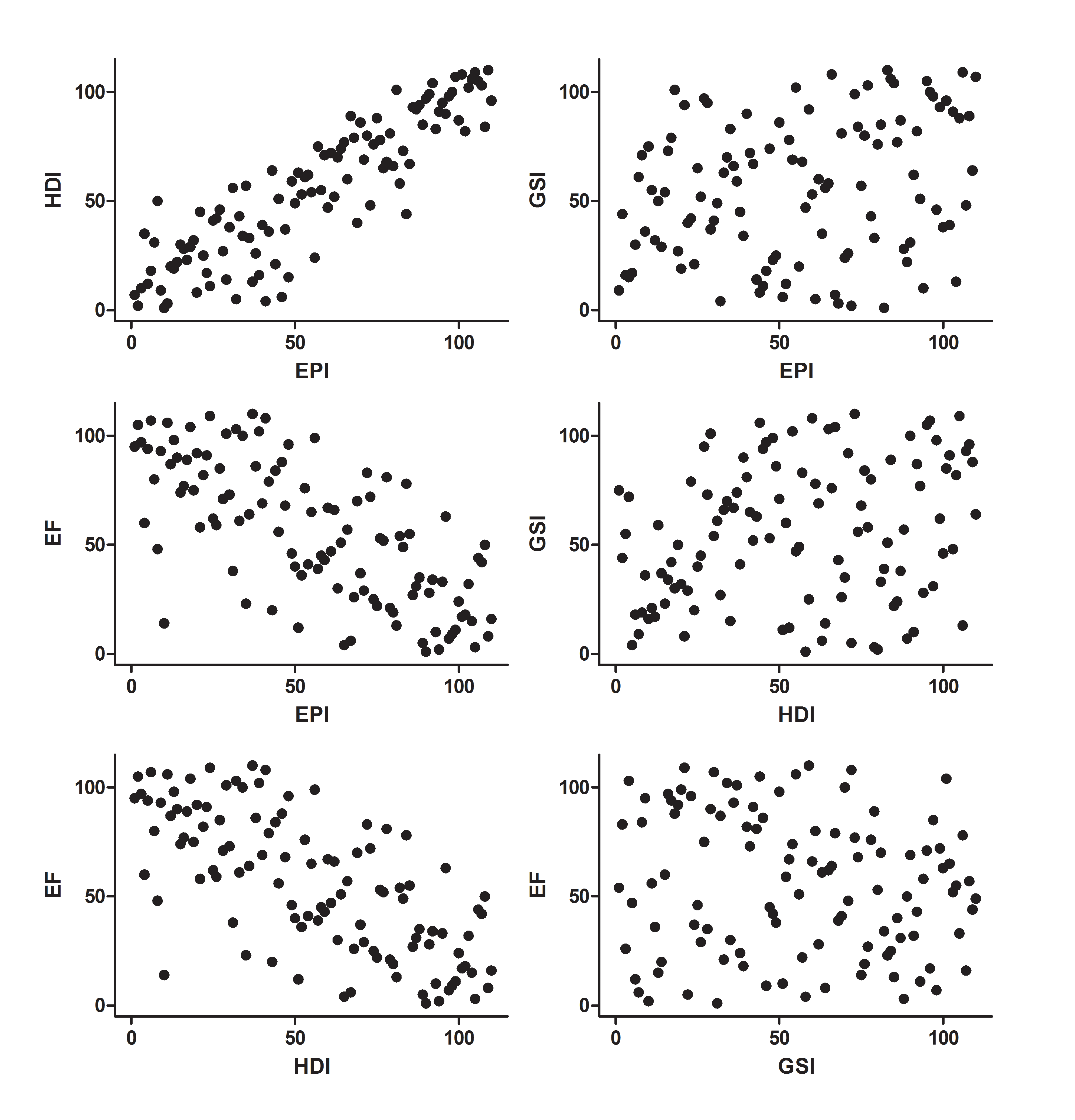

Supplement: Figure S1 — Rank correlations between existing environmental indicators. EPI = Environmental Performance Index, HDI = Human Development Index, GSI = Genuine Savings Index, EF = Ecological Footprint [reviewed in 8]. (0.83 MB TIF) [file pone.0010440.s012.tif]

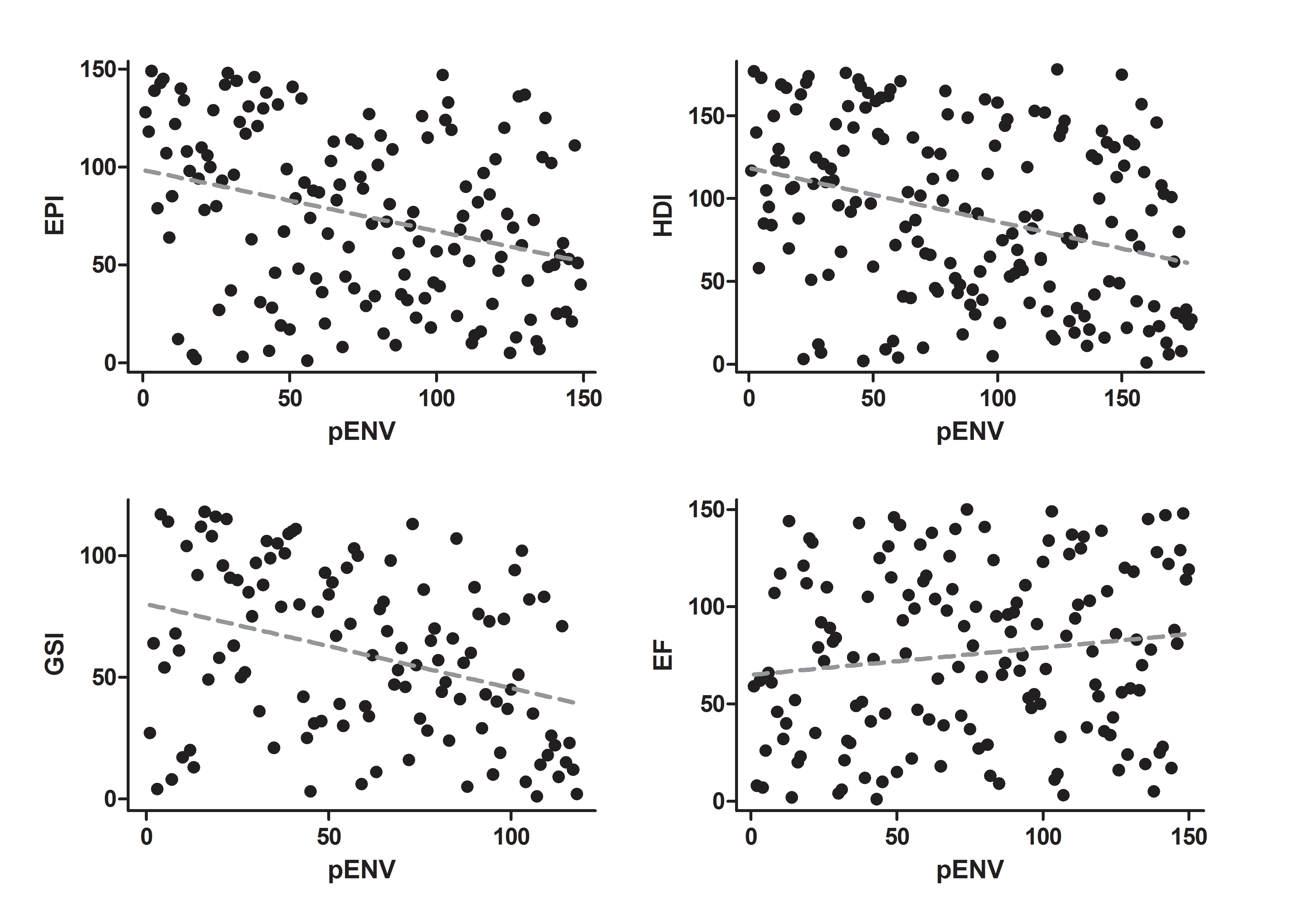

Supplement: Figure S2 — Rank correlations between proportional environmental impact (pENV) ranks and four existing environmental indicator ranks. EPI = Environmental Performance Index, HDI = Human Development Index, GSI = Genuine Savings Index, EF = Ecological Footprint [reviewed in 8]. (0.86 MB TIF) [file pone.0010440.s013.tif]

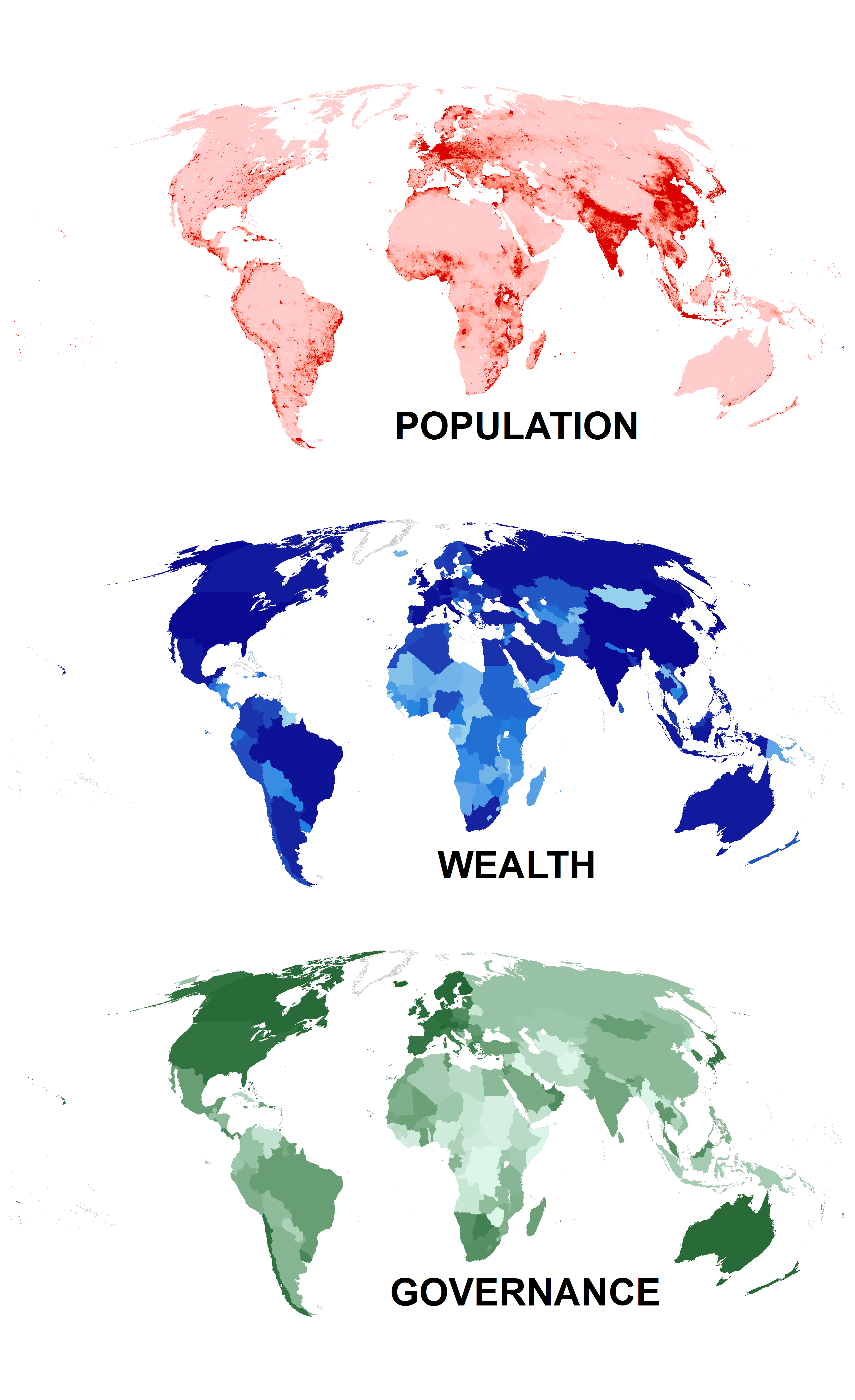

Supplement: Figure S3 — World distribution of socio-economic variables. Relative distributions of global human population (2005) (top panel: dark red = highest population), wealth rank (middle panel: dark blue = wealthiest based on purchasing power parity-adjusted Gross National Income) and governance quality rank (bottom panel: dark green = highest quality) among countries. (1.44 MB TIF) [file pone.0010440.s014.tif]

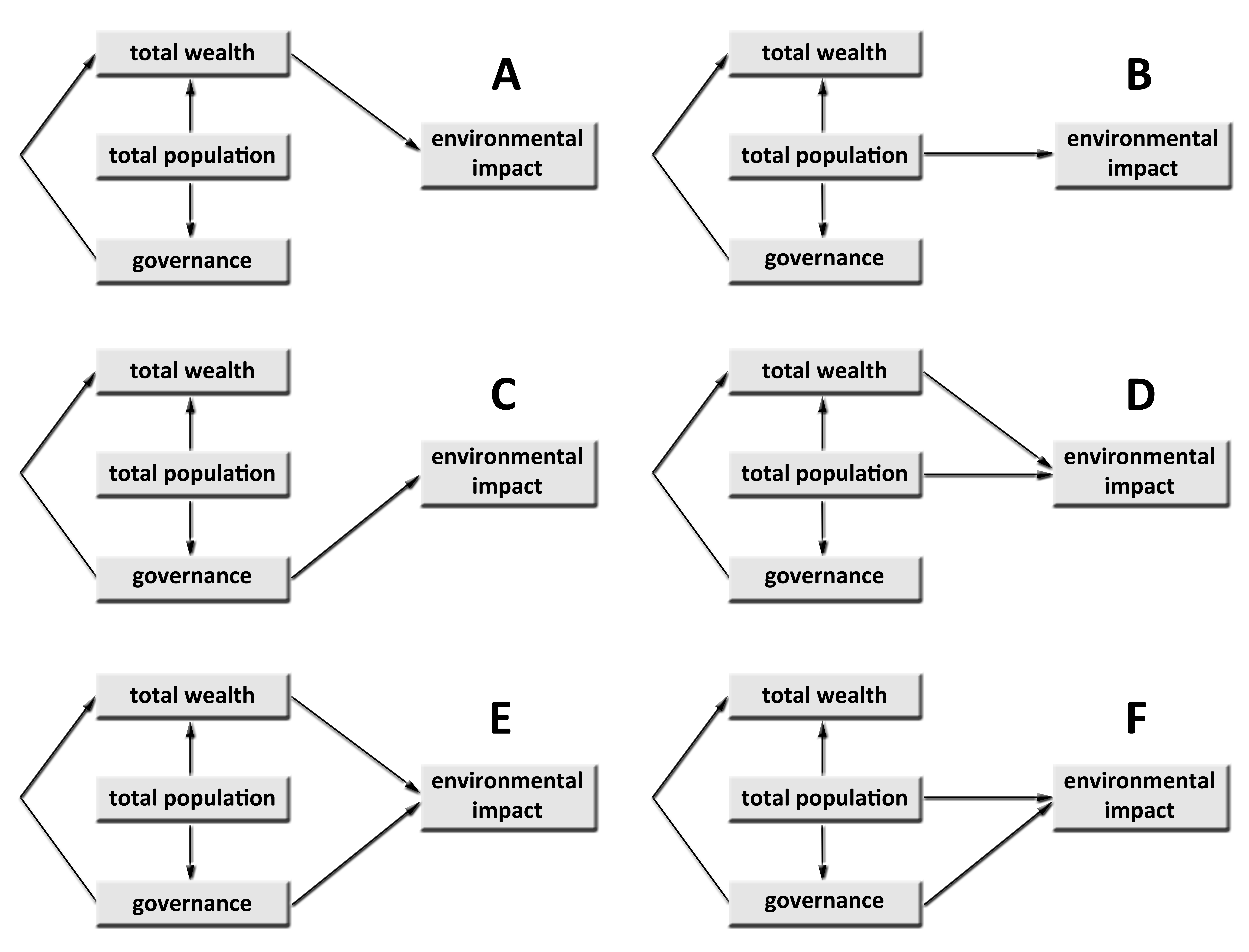

Supplement: Figure S4 — Path diagrams for the seven competing structural equation models A to G. Single-headed arrows represent hypothesized direct effects of one variable on another. (1.90 MB TIF) [file pone.0010440.s015.tif]
